# Serum Glycoproteomics and Identification of Potential Mechanisms Underlying Alzheimer's Disease

**DOI:** 10.1155/2021/1434076

**Published:** 2021-12-11

**Authors:** Naphatthakarn Kerdsaeng, Sittiruk Roytrakul, Suwannee Chanprasertyothin, Piangporn Charernwat, Sirintorn Chansirikarnjana, Piyamitr Sritara, Jintana Sirivarasai

**Affiliations:** ^1^Doctor of Philosophy Programme in Molecular Medicine, Faculty of Science & Faculty of Medicine Ramathibodi Hospital, Faculty of Medicine Siriraj Hospital & Faculty of Dentistry & Faculty of Tropical Medicine, Mahidol University, Bangkok, Thailand; ^2^Functional Ingredients and Food Innovation Research Group, National Center for Genetic Engineering and Biotechnology, National Science and Technology Development Agency, Pathum Thani, Thailand; ^3^Research & Innovation, Faculty of Medicine Ramathibodi Hospital, Mahidol University, Bangkok, Thailand; ^4^Department of Medicine, Faculty of Medicine Ramathibodi Hospital, Mahidol University, Bangkok, Thailand; ^5^Graduate Program in Nutrition, Faculty of Medicine Ramathibodi Hospital, Mahidol University, Bangkok, Thailand

## Abstract

**Objectives:**

This study compares glycoproteomes in Thai Alzheimer's disease (AD) patients with those of cognitively normal individuals.

**Methods:**

Study participants included outpatients with clinically diagnosed AD (*N* = 136) and healthy controls without cognitive impairment (*N* = 183). Blood samples were collected from all participants for biochemical analysis and for Apolipoprotein E (*APOE*) genotyping by real-time TaqMan PCR assays. Comparative serum glycoproteomic profiling by liquid chromatography-tandem mass spectrometry was then performed to identify differentially abundant proteins with functional relevance.

**Results:**

Statistical differences in age, educational level, and *APOE* ɛ3/ɛ4 and ɛ4/ɛ4 haplotype frequencies were found between the AD and control groups. The frequency of the *APOE* ɛ4 allele was significantly higher in the AD group than in the control group. In total, 871 glycoproteins were identified, including 266 and 259 unique proteins in control and AD groups, respectively. There were 49 and 297 upregulated and downregulated glycoproteins, respectively, in AD samples compared with the controls. Unique AD glycoproteins were associated with numerous pathways, including Alzheimer's disease-presenilin pathway (16.6%), inflammation pathway mediated by chemokine and cytokine signaling (9.2%), Wnt signaling pathway (8.2%), and apoptosis signaling pathway (6.7%).

**Conclusion:**

Functions and pathways associated with protein-protein interactions were identified in AD. Significant changes in these proteins can indicate the molecular mechanisms involved in the pathogenesis of AD, and they have the potential to serve as AD biomarkers. Such findings could allow us to better understand AD pathology.

## 1. Introduction

Alzheimer's disease (AD) or other forms of dementia have been reported in approximately 44 million people worldwide. AD is the most common cause of dementia, accounting for an estimated 60% to 80% of cases [1]. The population of Americans aged 65 and older is projected to grow from 56 million in 2020 to 88 million by 2050 [2]. The percentage of people with Alzheimer's dementia increases with age: 5.3% in people aged 65 to 74, 13.8% in people aged 75 to 84, and 34.6% in people aged 85 and older [3]. In China, the age-associated prevalence of AD is 3.2% in individuals over 60 years old, and it is predicted to increase from 3.8 to 6.2% in the next 5 years [4]. The prevalence of dementia in Thailand for people aged 45 years and above is 2.4% with AD being the most common type of dementia (75.0%) [5]. AD involves many factors and incorporates many hypotheses. A large number of human studies and animal models have described many of these factors and hypotheses including amyloid *β* (A*β*), tau, cholinergic neuron damage, calcium dyshomeostasis and pathological calcium signaling, oxidative stress, inflammation, apoptotic signals, and other intracellular signaling pathways.^6–8^ Transgenic mice were used to identify precursors to plaque formation and how the aggregation of A*β* is crucial to its toxicity [6]. The amyloid cascade hypothesis predicts that tau hyperphosphorylation occurs as a downstream consequence of A*β* accumulation [6]. In addition, the mechanisms involved in beta-amyloid-mediated inflammation act as an immune stimulus for glial/macrophage activation. Using immunocytochemistry to examine numerous cytokines including interleukin- (IL-) 1alpha, IL-1beta, IL-6, tumor necrosis factor- (TNF-) alpha, and macrophage chemotactic protein- (MCP-) 1, only IL-1beta was found to be induced in reactive astrocytes surrounding beta-amyloid deposits in 14-month-old transgenic Tg2576 mice [7]. A Taiwanese study compared amyloid beta peptide 42 (A*β*42) and tau levels in plasma samples using ultrasensitive immunomagnetic reduction assays, and they showed differences in plasma tau and A*β*42 levels. Levels of both proteins were higher in AD than in healthy, nondemented control subjects [8]. These findings and others support the use of plasma A*β*42 and tau as biomarkers in the clinical assessment of AD [8]. The underlying rationale of using core pathological proteins, including A*β*42, A*β*40, tau, tau phosphorylated at threonine-181 (Thr181P), inflammatory cytokines, oxidized proteins, and proteins in signaling pathways, has been widely stated. Proteomics is an emerging method for the identification of proteins, investigation of posttranslational modification, and determination of complex interactions of proteins in cellular systems (including their structure, function, and localization) [9]. Comprehensive proteomic analysis of human plasma or serum is a strategy used to identify biomarkers that underlie pathophysiology thereby promoting advances in diagnostic profiling, disease monitoring, and treatment [10]. Comprehensive quantitative analyses of proteins in AD and controls by high-resolution two-dimensional liquid chromatography and tandem mass spectrometry (LC/LC-MS/MS) identified differentially expressed proteins in AD (26 downregulated and 4 upregulated). These proteins were related to known pathways of mitochondrial dysfunction, fatty acid beta oxidation, and advanced glycation end products (AGEs) and their receptors (RAGEs) [11].

Glycoproteomics focuses on studying the profile of glycosylated proteins, and analysis involves tryptic glycopeptide enrichment following LC-MS/MS. Protein glycosylation is a complex posttranslational modification that is associated with the biological activity and function of glycoproteins [12]. Moreover, alterations in glycosylation profiles are associated with many diseases, including cancer, inflammation, and neurodegenerative diseases [13]. A glycoproteome profiling study characterizing protein N-glycosylation in human AD and control brains produced significant findings. Multiple dysregulated n-glycosylation-affected processes and pathways were observed in an AD brain, including extracellular matrix dysfunction, neuroinflammation, synaptic dysfunction, cell adhesion alteration, lysosomal dysfunction, endocytic trafficking dysregulation, endoplasmic reticulum dysfunction, and cell signaling dysregulation [14].

However, analysis of glycosylation of AD-related proteins in serum or plasma remains to be fully explored. Here, we describe a glycoproteomic study comparing Thai AD patients with normally cognitive individuals. This study describes AD serum-enriched glycoproteins, which have the potential to be used as diagnostic and/or prognostic markers.

## 2. Materials and Methods

### 2.1. Study Design and Subjects

This study included outpatients with dementia due to AD and healthy controls without cognitive impairment, who were all fluent in Thai. A total of 136 AD individuals (aged ≥65 years) were recruited from the geriatric and psychiatry (memory) outpatient clinics, Faculty of Medicine Ramathibodi Hospital, Mahidol University, between 2011 and 2013. All patients were diagnosed by impairments in memory, thinking, and behavior, which decreased each person's ability to function independently in everyday life. Clinical assessments and biomarker evidences were carried out in some AD cases to provide additional diagnostic certainty. Such assessments included neuropsychological testing, specialized structural neuroimaging with magnetic resonance imaging (presence of medial temporal lobe atrophy), and cerebrospinal fluid analysis of tau/A-beta [15]. We further accessed medical records and contacted patients who were diagnosed with AD. The controls were participants from the Electricity Generating Authority of Thailand (EGAT) study conducted in 2013 (*N* = 183, age ≥ 55 years) with normal cognitive function (evaluated by Montreal Cognitive Assessment (MoCA) ≥ 26). Exclusion criteria included history of head injury, chronic metabolic diseases, severe auditory or visual sensory impairment, severe concurrent medical illness such as severe heart diseases and malignant tumors, or major psychiatric disorders such as major depression, bipolar disorder, and schizophrenia. Informed consent was obtained from all subjects, and all procedures for this study were approved by the Ethics Committee on Human Rights Related to Research Involving Human Subjects, Faculty of Medicine Ramathibodi Hospital, Mahidol University.

### 2.2. Biochemical Measurements

Venous blood samples were collected in the morning after an overnight fast (12 hours), and serum samples were separated and stored at −80°C for subsequent analysis. Glycated hemoglobin (HbA1c), fasting blood glucose (FBG), total cholesterol, triglyceride (TG), high-density lipoprotein (HDL) cholesterol, low-density lipoprotein (LDL) cholesterol, total protein, albumin, urea nitrogen, and creatinine concentrations were measured using automated methods (Cobas-Mira, Roche, Milan, Italy). The levels of plasma folate and vitamin B12 were determined by electrochemiluminescence immunoassays (ECLIA).

### 2.3. *APOE* Genotyping

Genomic DNA was extracted from whole blood in EDTA-coated tubes by a standard phenol-chloroform extraction procedure and frozen at −20°C until use. The DNA concentration was measured using a NanoDrop 2000™ Spectrophotometer (Thermo Fisher Scientific, Massachusetts, USA). The *APOE* single-nucleotide polymorphisms (SNPs) (rs429358 and rs7412) were genotyped using TaqMan® real-time polymerase chain reactions (real-time PCR). TaqMan SNP assays, designed by Applied Biosystems, are delivered as 20× or 40× single tube mixtures (188 *μ*L) of forward and reverse primers (900 *μ*M) and two reporter probes (200 *μ*M). The 5′ end of each probe is linked to different fluorescent allele-specific dyes: fluorescein amidite (FAM) is allele 2 specific, while VIC is the reporter for allele 1. The 2× TaqMan universal PCR master mix (Applied Biosystems) used contains AmpliTaq Gold® DNA polymerase, dNTPs, and a passive internal reference based on proprietary ROX dye. To prepare the reaction mix to amplify 96 samples in a 96-well plate format, 15 *μ*L of 20× working stock of the SNP Genotyping Assay (or 7.5 *μ*L of 40× stock) was added to 285 *μ*L of 2× universal master mix diluted with 200 *μ*L of distilled water. After vortexing, 10 *μ*L of the mixture was transferred into each well of a 96 reaction plate before adding 20 ng of wet genomic DNA. The plate was sealed and inserted into the One Step Applied Biosystems real-time PCR machine. PCR temperature was kept on hold for 10 minutes at 95°C, then reduced to 92°C for 15 seconds (denaturation), and further reduced for annealing and extension stages to 60°C for 1 minute for 40 cycles.

### 2.4. Isolation of Glycoprotein from Serum Samples Using Concanavalin A

Protein concentration of serum samples was determined according to the Lowry protein assay using bovine serum albumin (BSA) as the standard [16]. Five hundred micrograms of protein from each sample was taken for glycoprotein enrichment using a Pierce glycoprotein isolation kit, ConA, and then desalted using the Zeba™ Desalt spin column according to the manufacturer's protocol. To reduce disulfide bond, 10 mM dithiothreitol in 10 mM ammonium bicarbonate was added to 5 *μ*g of purified glycoproteins, and reformation of disulfide bonds in the glycoproteins was blocked by alkylation with 30 mM iodoacetamide in 10 mM ammonium bicarbonate. The glycoprotein samples were digested with 200 ոg of sequencing grade porcine trypsin (Promega; Mannheim, Germany) for 12 h at 37°C. The tryptic peptides were dried using a speed vacuum concentrator and kept at -80°C for further mass spectrometric analysis.

### 2.5. Protein Quantitation and Identification by LC-MS/MS

Tryptic peptide samples were resuspended in 0.1% formic acid before injecting into an Ultimate3000 Nano/Capillary LC System (Thermo Scientific, UK) coupled to a Hybrid quadrupole Q-TOF impact II™ (Bruker Daltonics) equipped with a nanocaptive spray ion source. Briefly, peptides were enriched on a *μ*-precolumn (300 *μ*m i.d. ×5 mm with C18 PepMap 100, 5 *μ*m, 100 A, Thermo Scientific, UK) and separated on a 75 *μ*m I.D. ×15 cm column packed with Acclaim PepMap RSLC C18 (2 *μ*m, 100 Å, nanoViper, Thermo Scientific, UK). Solvent A and B contain 0.1% formic acid in water and 0.1% formic acid in 80% acetonitrile, respectively. A gradient of 5–55% solvent B was used to elute the peptides at a constant flow rate of 0.30 *μ*L/min for 30 min. Electrospray ionization was carried out at 1.6 kV using the CaptiveSpray. Mass spectra (MS) and MS/MS spectra were obtained in the positive-ion mode over the range (*m*/*z*) 150–2200 (Compass 1.9 software, Bruker Daltonics). The LC-MS analysis of each sample was done in triplicate.

MaxQuant 1.6.1.12 was used to quantify the proteins in individual samples using the Andromeda search engine to correlate MS/MS spectra with the UniProt *Homo sapiens* database [16]. The following parameters were used for data processing: maximum of two miss cleavages, mass tolerance of 20 ppm for the main search, trypsin as the digesting enzyme, carbamidomethylation of cysteines as fixed modification, and the oxidation of methionine and acetylation of the protein N-terminus as variable modifications. Only peptides with a minimum of seven amino acids, as well as at least one unique peptide, were required for protein identification. Only proteins with at least two peptides, and at least one unique peptide, were considered being identified and used for further data analysis.

All differentially expressed proteins were analyzed for their intersections among the different sample groups using jvenn [17]. Gene Ontology annotation, including biological process, cellular component, and molecular function, was performed using Panther (http://www.pantherdb.org). The identified proteins were simultaneously submitted to the Search Tool for Interacting Chemicals (STITCH) (http://stitch.embl.de) to search for understanding of cellular functions and interactions between proteins and small molecules.

### 2.6. Statistical Analysis

All statistical analyses were performed with SPSS Statistics for Windows, version 22.0 (IBM SPSS Statistics for Windows, version 23.0. Armonk, NY: IBM Corp). Data are presented as the mean ± standard deviation. Allele and genotype differences between groups and deviations from the Hardy-Weinberg equilibrium were tested by chi-square tests.

## 3. Results

### 3.1. Demographic and Clinical Characteristics of Participants

The control group consisted of healthy individuals with normal cognitive function (with a mean age of 56.52 ± 4.13 years; more than 60% were female (Table 1)). A normal Montreal Cognitive Assessment (MoCA) score is considered to be ≥26. The mean MoCA of the control group was 28.20, and 86.9% of participants had >12 years of education. Biochemical analysis related to glycemic parameters, lipid profile, kidney, and thyroid functions was in the reference ranges. For the AD group, the mean age was 78.08 ± 7.47 years; 77.95% were female (*n* = 136) and 58.1% have educational level ≤ 12 years, as shown in Table 2. Most patients reported no family history of AD, and their diagnoses were confirmed by physicians in the geriatric and psychiatry (memory) outpatient clinic. Similar to the control group, blood biochemistry tests in the AD group were in normal ranges.

### 3.2. Distributions of APOE Haplotypes and Alleles in Control and AD Groups

Six and five *APOE* haplotypes were detected in the control and the AD groups, respectively. The most common *APOE* haplotypes in the control and AD groups was ɛ3/ɛ3. The ɛ2/ɛ2 was a haplotype not found in the AD group (Table 3). Statistical differences in *APOE* ɛ3/ɛ4 and ɛ4/ɛ4 haplotype frequencies were found between both groups. The frequency of the ɛ4 allele was significantly higher in the AD group than in the control group, whereas the ɛ3 allele frequency was significantly higher in the control group than in the AD group (*P* < 0.05).

### 3.3. Proteomic Analysis

**Figure 1:**
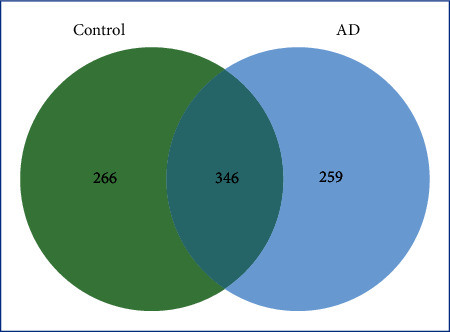
Venn diagrams displaying the number of identified unique glycoproteins for control and AD groups.

**Figure 2:**
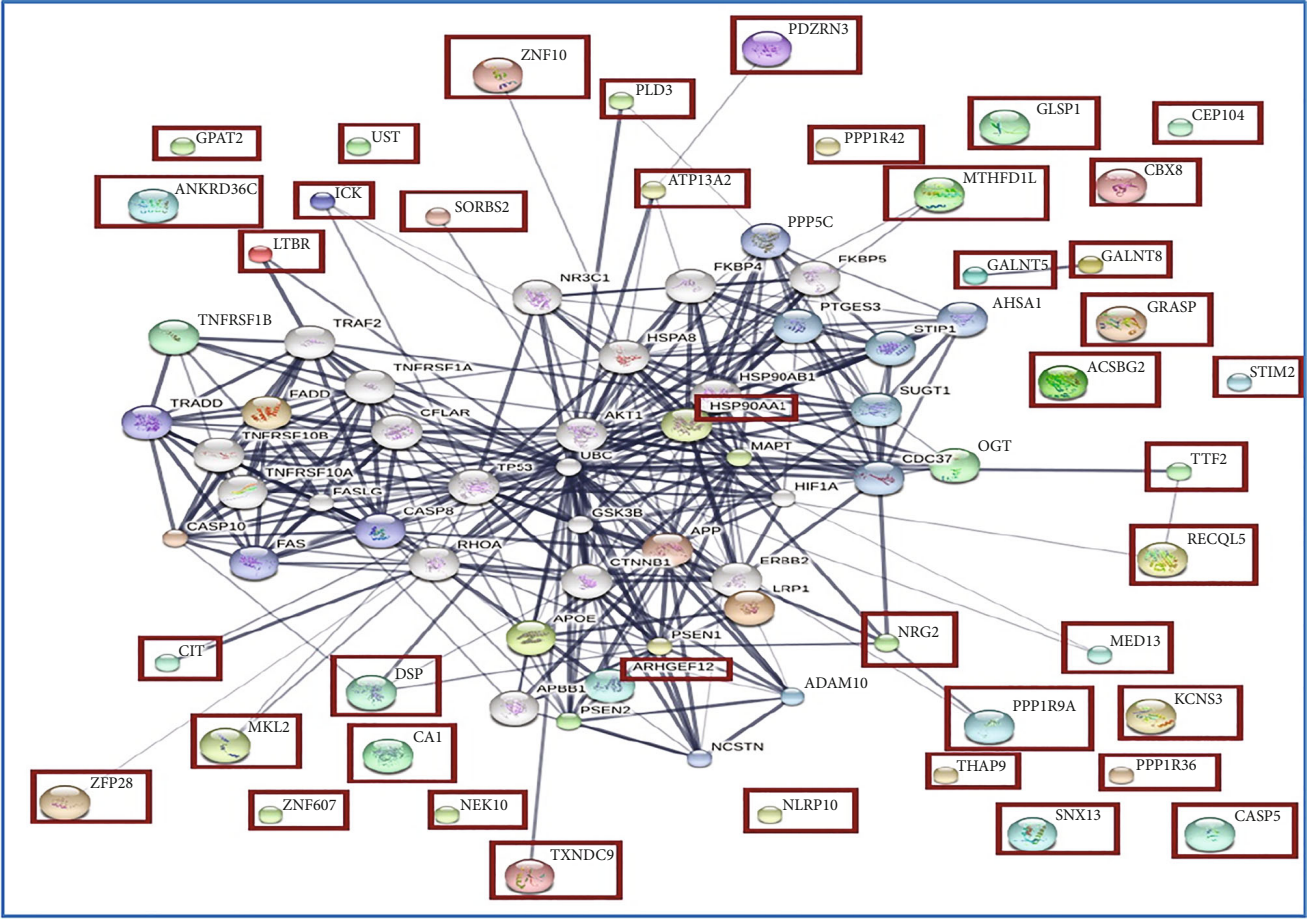
Protein-protein interaction identified from serum of the AD group. Stronger associations are represented by thicker lines. Protein-protein interactions are shown in grey, chemical-protein interactions in green, and interactions between chemicals in red.

Proteomic analysis of serum protein patterns in control and AD groups are described in Tables 1–6 and Figures 1 and 2. Approximately 600 proteins from each of the control and AD groups were identified at a false discovery rate of 1%. Comparative proteomic analysis was performed between sera of the two groups with 42 upregulated glycoproteins in Table 3 and 287 downregulated glycoproteins in Table 4 (for all proteins in this group, see Supplementary Table 1).  Table 5 shows the classification of upregulated proteins as follows: protein-binding activity modulator (16.7%), gene-specific transcriptional regulator (12.5%), metabolite interconversion enzyme (12.5%), and others. Moreover, categories with the highest percentages of downregulated proteins were interconversion enzymes (13.7%), protein-modifying enzymes (12.4%), proteins used in nucleic acid metabolism (12.4%), and others (Table 5). Further analysis using the Universal Protein Resource (UniProt) identified the top five upregulated glycoproteins including A disintegrin and metalloproteinase with thrombospondin motifs 8 (ADAMTS8), ubiquitin-fold modifier-conjugating enzyme 1 (UFC1), kinesin-like protein KIF28P (KIF28P), protein Ras-related protein Rab-6A (RAB6A), and coagulation factor X (FX). The top five downregulated glycoproteins were C-type mannose receptor 2 (MRC2), FAS-associated factor 2 (FAF2), NAD-dependent protein lipoamidase sirtuin-4 (mitochondrial; SIRT4), ankyrin repeat domain-containing protein 13C (ANKRD13C), and brain-specific angiogenesis inhibitor 1-associated protein 2-like protein 1 (BAIAP2L1).

In total, 871 glycoproteins were identified, including 266 and 259 unique proteins in control and AD groups, respectively, as represented by a jvenn diagram (Figure 1). Further analysis was performed on the unique glycoproteins in AD patients. The classification and pathways of the identified glycoproteins were analyzed by the UniProt tool and PANTHER-gene list analysis (Table 6). According to molecular function, glycoproteins were classified as organic cyclic compound binding (26.0%), heterocyclic compound binding (26.0%), protein binding (24.7%), ion binding (5.5%), small molecule binding (5.5%), and carbohydrate binding (5.6%). For biological process, these glycoproteins were categorized as cellular process (32.3%), metabolic process (15.5%), biological regulation (15.1%), and others. In addition, relevant pathways were identified as Alzheimer disease-presenilin pathway (16.6%), inflammation pathway mediated by chemokine and cytokine signaling (9.2%), Wnt signaling pathway (5.1%), and apoptosis signaling pathway (5.1%), as described in Table 6.

Pathway analysis of the differentially expressed proteins in the AD group was performed using the STITCH database, and the generated protein-protein network is presented in Figure 2. The network indicated interactions among members of the 45 unique glycoproteins identified in the AD group with pivotal proteins in pathogenesis and prevention of AD including APP, MAPT, tau protein, PSEN1, PSEN2, and APOE. An interaction network comprising 346 interactions was generated. These interactions were ascribed to Gene Ontology biological processes, including necroptic process, activation of cysteine-type endopeptidase activity involved in apoptotic signal pathway, positive regulation of catalytic activity, proteolysis, and I-kappaB kinase/NF-kappaB signaling, and to Kyoto Encyclopedia of Genes and Genomes (KEGG) pathways including apoptosis, AD pathways, and TNF signaling pathways.

## 4. Discussion

A decline in cognitive function is partly predisposed by increasing age as seen in the prevalence of AD or other dementias worldwide [1, 2, 4], which is congruent with our findings. Proposed hypotheses involve age-related protein abnormalities and pinpointed protein dyshomeostasis in the aging brain [18, 19]. Our findings also indicate a correlation between low education and a high risk of AD dementia, which coincides with a previous meta-analysis study that found a higher incidence of dementia in people with a lower level of education [20]. A plausible explanation for the effect of education on the clinical expression rather than the pathologic course of AD or dementia has been postulated [21].

The possible pathophysiological mechanisms related to AD in the Thai population were investigated by considering glycoproteomes and *APOE* gene data. Multiple genetic and environmental risk factors are involved in AD pathogenesis. Findings from previous studies support a role for genetic determinants in AD in 90% of early-onset AD (EOAD) cases and 58–79% of late-onset AD (LOAD) cases [22]. Genetic polymorphism in *APOE* is the main genetic risk factor in LOAD, with increased risk of AD for carriers of the *ε*4 allele, whereas the *ε*2 allele conferred a decreased risk [23]. Significantly higher haplotypes and allele frequencies for *ε*4 heterozygotes (*APOE ε*3/*ε*4) and *ε*4 homozygotes (*APOE ε*4/*ε*4) were found in the AD group compared with the control group (Table 3). The prevalence of *APOE ε*4 carriers (42.7%) was in agreement with that for Asia (40.6%), but lower than those for Southern Europe (44.1%), Central Europe (54.8%), North America (58.5%), Northern Europe (64.8%) [23], and South America (54.8%) [24]. The effects of *APOE ε*4 on AD risk have been proposed to include the following: inhibition of A*β* clearance and promotion of A*β* aggregation, tau pathology and tau-mediated neurodegeneration, impairment of microglia-inflammation response, disturbance of lipid transport, loss of synaptic integrity and plasticity, abnormal glucose metabolism, and disruption of cerebrovascular integrity and function [25].

The final form of a gene product is the synthesized protein; therefore, the proteome is directly related to biological function and is responsive to physiological states and diseases. Several quantitative proteomic studies in AD have been carried out on patient-derived biological samples including the brain, cerebrospinal fluid (CSF), and plasma. Both brain and CSF samples are known as the representative biomarkers for neurodegenerative analysis because their changes directly reflect brain pathophysiology, neuronal damage, and disease progression [26, 27]. However, brain tissue and CSF biomarker are not practical for the next step of biomarker discovery since validation methods involve a large number of cases and different disease stages. Serum-based proteomic biomarkers provide several advantages including the following: (1) it is a more practical approach with identification of numerous proteins from all tissues, (2) it is inexpensive, and (3) it provides a more effective tool for future research and clinical diagnosis since it implements only a blood test [10].

To better elucidate mechanisms underlying AD, we investigated alterations in serum glycoproteins. Our results show both upregulated and downregulated glycoproteins in AD sera compared with control sera. A disintegrin and metalloproteinase with thrombospondin motifs 8 (ADAMTS8) was upregulated in AD patients. ADAMTS8 is an ADAMTS member, a unique family of the extracellular matrix (ECM) proteases found in mammals. These glycoproteins are involved in neuroplasticity and the development of chronic neurodegenerative disorders, including AD [25]. The possible functions of ADAMTS proteases in AD might be linked to Reelin, a glycoprotein essential for brain development and function [28]. Decreased Reelin activity after ADAMTS digestion may lead to amyloid-*β* deposition, Tau phosphorylation, and tangle formation in the hippocampal formation through disturbance of phosphatidylinositol-3-kinase (PI3K), protein kinase B (PKB/Akt), and glycogen synthase kinase 3*β* (GSK3*β*) signaling [28–31]. Ubiquitin-fold modifier-conjugating enzyme 1 (UFC1) was upregulated in our AD cohort. This enzyme is a component of UFM1 (ubiquitin-fold modifier 1), which is responsible for UFMylation and required for E1 activating enzymes, E2 conjugating enzymes, and an E3 ligase [32]. UFMylation is tightly related to endoplasmic reticulum (ER) stress and is involved in protein folding and secretion. The consequences of ER stress may lead to the amyloid cascade, tau phosphorylation, and synaptic dysfunction [33]. Kinesin-like protein KIF28P (KIF28P), a kinesin-3 vesicle transport protein [34], was also upregulated in the AD group. Data related to KIF28P and AD are limited; however, evidence indicates that KIF1A, belonging to the kinesin-3 family, is a neuron-specific kinesin [34]. It has a primary role in the anterograde transport of beta-secretase (BACE1), as determined by abnormal accumulation of BACE1 at presynaptic terminals in the postmortem AD brain [35]. The elevation of local BACE1 levels can promote the generation of A*β*, contributing to the development of AD [36]. Ras-related protein Rab-6A (RAB6A) is a low molecular weight GTPase that belongs to the Ras superfamily and regulates retrograde Golgi-ER trafficking, transport of early endosomes and recycling endosomes to the trans-Golgi network, and vesicle exocytosis [37]. Previous studies reported significant associations of RAB6A with AD pathology, including increased levels in AD brains [38], which affects APP trafficking and A*β* generation [39] and modulates the AD-related unfolded protein response [38]. Coagulation factor X (FX), a vitamin K-dependent glycoprotein, was also upregulated in AD sera. FX plays a central role in the activation of prothrombin to thrombin in the presence of calcium ions in the blood coagulation cascade [40]. Thrombin is a key mediator of coagulation and inflammation via proteolytic and receptor-mediated mechanisms. Thrombin acts as a vascular-derived inflammatory protein that can activate both microglia and astrocytes, resulting in release of inflammatory proteins, reactive oxygen species, and proteases [41]. Induction of proinflammatory cytokines, including IL-1*β*, IL-6, and TNF-*α*, by thrombin is also evidence for cerebral vasculature damage contributing to neuroinflammation and neuronal injury in AD [42].

Among the downregulated glycoproteins found in our study, C-type mannose receptor 2 (MRC2) is from the family of endocytic receptors. MRC2 is involved in the intracellular collagen degradation pathway in biological and pathological processes [43]. The endocytic pathway (EP) has been described to have early endosomal abnormalities in subtypes of AD, resulting in amyloid beta generation and APOE dysfunction [44]. MRC2 is also downregulated in aging human microglia [45]. Another downregulated protein found in this study is FAS-associated factor 2 (FAF2). FAF2 has an important role in endoplasmic reticulum-associated degradation, which mediates ubiquitin-dependent degradation of misfolded ER proteins [46]. Significant underexpression of *FAF2* was determined in the frontal cortex of AD patients [47]. SIRT4 was downregulated in AD patients. The potential role of sirtuins in AD and other neurodegenerative disorders has been described. Reductions of SIRT1 and SIRT3 mRNA/protein levels were observed in AD brains correlating with the stage/duration of the disease [48]. In addition, increased expression of *SIRT4* after short-term treatment with extracellular A*β*1–42 oligomers indicates a more complex association between APP/A*β* and SIRTs [49]. Ankyrin repeat domain-containing protein 13C (ANKRD13C) was also downregulated in AD. This glycoprotein acts as a molecular chaperone for G protein-coupled receptors (GPCRs), regulating their biogenesis and exit from the ER [50]. Alterations of GPCRs can lead to interaction between GPCRs and *β*-site APP cleaving enzyme 1 (BACE1), a key secretase in AD pathogenesis [51]. Brain-specific angiogenesis inhibitor 1-associated protein 2-like protein 1 (BAIAP2L1), also known as insulin receptor tyrosine kinase substrate (IRTKS), is a novel regulator of the insulin network [52]. IRTKS-deficient mice exhibit insulin resistance, including hyperglycemia, hyperinsulinemia, glucose intolerance, decreased insulin sensitivity, and increased hepatic glucose production [53]. Insulin dysregulation can contribute to AD through a role in proteostasis. Insulin resistance can lead to abnormal clearance of amyloid *β* peptide and phosphorylation of tau, along with effects on vasoreactivity, lipid metabolism, and inflammation [54].

Further analysis of 259 unique glycoproteins in the AD group categorized the main proteins as binding activity (41.7%) for molecular function, cellular process (27.5%) for biological process, and Alzheimer's disease-presenilin pathway (16.6%) for pathway. Previous studies have shown main plasma proteins classified as acute-phase response (upregulated) and platelet degranulation (downregulated) for biological process. It also classified them as complement-component C1q binding (upregulated) and serine-type endopeptidase inhibitor activity (downregulated) for molecular function [55]. To get a general overview of serum glycoprotein interactions in AD and to search for the most affected pathways involving the candidate proteins, analysis with the STITCH database was performed (Figure 2). Three KEGG pathways were highlighted: apoptosis, AD pathway, and TNF signaling pathway. For apoptosis pathways, protein networks with MAPT, APOE, APP, PSEN1, and PSEN2 included a disintegrin and metalloproteinase (ADAM10), LDL receptor-related protein (LRP1), polyamine-transporting ATPase 13A2 (ATP13A2), proneuregulin-2, membrane-bound isoform (NRG2), Desmoplakin (DSP), Rho guanine nucleotide exchange factor 12 (ARHGEF12), citron Rho-interacting kinase (CIT), and protein phosphatase 1 regulatory subunit 9A (PPP1R9A). ADAM10 is the main *α*-secretase that cleaves APP in the nonamyloidogenic pathway, and it plays a critical role in reducing the generation of A*β* peptides [56]. LRP1 regulates A*β* metabolism in the brain and brain homeostasis. The impairment of these processes results in AD pathology [57]. ATP13A2 is a late endolysosomal transporter. Loss of function of this protein results in lysosomal deficiency as a consequence of impaired lysosomal export of the polyamines spermine/spermidine, and it is implicated in neurodegenerative disorders [58]. Another glycoprotein, NRG2, is a neuregulin (NRG), a family of epidermal growth factor- (EGF-) related proteins. Dysregulation of this glycoprotein can lead to abnormal synaptic plasticity in the hippocampus and AD development [59]. DSP is a component of the adherence junction complex and plays a role in endothelial cell (EC) shear stress and/or hypoxia regulation. Higher expression of DSP in intracranial ECs suggests stronger cell-cell contact between intracranial ECs compared with extracranial ECs, which affects the blood-brain barrier of the cerebral microvasculature [60]. Proteome-based assessment of plasma biomarkers in 511 subjects with AD along with other neurodegenerative diseases and normal older people showed that DSP was upregulated in the AD group [61]. ARHGEF12 plays a role in the regulation of RhoA GTPase and in the RhoA/RhoA kinase pathway [62]. Impairment of these Rho GTPases contributes to the increase in A*β* resulting in neurotoxicity, as seen in AD [63]. CIT is a tissue-specific Ser/Thr kinase that targets the Rho-Rac-binding protein, citron. In AD brains, dysregulation of Rho-Rac1-GTPase signaling contributes to synaptic degeneration, amyloid precursor protein (APP) processing, and an increase in tau phosphorylation [64]. PPP1R9A or Neurabin-1 plays an important role in synaptic structure and function and neurite formation. In addition, abnormal protein phosphatase activity can cause hyperphosphorylation and lead to the aberrant accumulation of A*β* plaques. It can also lead to the intracellular formation of hyperphosphorylated tau protein together with synaptopathology in AD [65].

Interaction of proteins potentially related to apoptosis pathways were found in the AD group, including caspase 10 (CASP10) and FAS-associated death domain protein (FADD) (Figure 2). CASP10 is an apical caspase. When a death signal triggers an apoptotic pathway, apical caspases are activated that then activate effector caspases by proteolytic cleavage, which leads to apoptotic cell death [66]. Human CASP10 is highly homologous to caspase-8 and the role of CASP10 is also FADD-dependent; however, its role in the extrinsic apoptotic cascade is still not clear [66]. A previous study of AD cases revealed that caspase-8 and its downstream effector, caspase-3, are involved in synaptic plasticity, learning and memory, and control of microglia proinflammatory activation and associated neurotoxicity, which is indicated in AD pathology [67]. FADD is an adaptor protein involved in initiating apoptosis and in extrinsic/death receptor-mediated apoptosis. However, along with serine/threonine kinases, it is also responsible for the initiation of necroptosis [68]. A study of the possible contribution of apoptotic processes and other pathological cascades in the degeneration of cholinergic neurons in AD indicated that FADD apoptotic signaling may be triggered within basal forebrain cholinergic neurons in AD [69]. KEGG pathway analysis in this study indicated the TNF signaling pathway to be involved in protein-protein interaction. Tumor necrosis factor receptor superfamily member 1B (TNFRSF1B), also known as TNFR2, was the main glycoprotein reported in AD patients. Transgenic mice expressing human TNFR2 in primary oligodendrocytes showed that specific activation of TNFR2 rescues neurons and oligodendrocytes from oxidative stress and promotes oligodendrocyte differentiation and myelination [70]. Another gene-targeting study to delete TNFR2 in an AD transgenic mouse model found overexpression of TNFR2 ameliorated progression of the disease. These findings reinforce a neuroprotective function of TNFR2 in AD pathology [71]. We identified other glycoprotein interactions potentially related to AD pathology. The E3 ubiquitin-protein ligase, PDZRN3, promotes endocytosis and lysosomal degradation and is involved in the protein ubiquitination pathway [72]. Crosstalk between the enzymes that regulate protein ubiquitination and the toxic proteins, Tau and A*β*, were highlighted for next-generation therapeutics strategies [73]. The 5′-3′ exonuclease PLD3 (PLD3), also found in the protein interaction analysis, regulates inflammatory cytokine responses via degradation of nucleic acids by reducing the concentration of single strand DNA able to stimulate Toll-Like Receptor 9 (TLR9) [74]. Stimulation of innate immunity via TLR9 in a transgenic mouse model of AD was highly effective at reducing the parenchymal and vascular amyloid burden, along with A*β* oligomers [75]. Thioredoxin domain-containing protein 9 (TXNDC9) or phosducin-like protein 3 (PHLP3) has ATP binding activity. TXNDC9 regulates the ATPase activity of chaperonin T-complex protein 1 complex, a key complex for protein folding, and it diminishes actin and tubulin folding [76]. Misfolded tubulin aggregates, accumulates, and tangles in plaques, a hallmark of AD. Mass spectrometry analysis identified significant decreases of human brain t-complex polypeptide 1 and *β* 1 tubulin in the temporal, frontal and parietal cortex and thalamus of patients with AD [77].

In this study, we have identified many proteins associated with AD; however, there are several important limitations. The first is differences in age and education between control and AD groups. Higher age and lower education were reported as having a greater risk for dementia, but the outcomes are not always consistent [78]. Nevertheless, obtaining a larger number of cases and healthy controls for stratified age groups, educational level, and APOE haplotypes will be beneficial for clarifying differences in protein profiles. This is an essential step for both effective and achievable clinical application. Another limitation involves the blood-brain barrier that may contribute to some obstacles of interpretation between proteins in the blood and their association in brain. The last limitation is that we did not measure relevant neurodegenerative biomarkers such as tau, A*β* 40, and 42 proteins in AD cases. Future work is necessary to evaluate these hallmarks and their impact on the protein profiles.

## 5. Conclusions

Our mass spectrometry-based proteomic study of AD patients and normally cognitive controls is an efficient and comprehensive way to identify thousands of proteins in serum samples. These protein changes can clarify some gaps in our understanding about the molecular mechanisms that underlie the pathogenesis of AD. Network glycoproteome analysis not only reveals a highly reproducible and integrated window into the complex biochemical and cellular alterations in the serum of individuals with AD but also fulfills many clinical needs in AD diagnostics, disease monitoring, and therapeutics.

## Figures and Tables

**Table 1:** Characteristics and clinical parameters of the controls (normal cognitive function) and the AD group.

Characteristics	Normal cognitive function (*N* = 183)	Alzheimer's disease (AD) (*N* = 136)
Age, years	56.52 ± 4.13	78.08 ± 7.47^∗^
Gender		
Male	50 (27.3)	30 (22.1)
Female	133 (72.7)	106 (77.9)
Body mass index (BMI) (kg/m^2^)	24.82 ± 3.41	22.91 ± 3.24
Blood pressure (mmHg)		
Systolic blood pressure	124.65 ± 9.85	110.39 ± 7.89
Diastolic blood pressure	80.92 ± 9.88	77.45 ± 10.23
Educational level		
≤12 years	24 (13.1%)	79 (58.1)^∗^
>12 years	159 (86.9%)	57 (41.9)
FBG (mg/dL)	102.48 ± 28.42	102.5 ± 36.7
Total cholesterol (mg/dL)	202.00 ± 36.45	186.74 ± 40.15
LDL-cholesterol (mg/dL)	132.24 ± 42.12	115.61 ± 36.42
HDL-cholesterol (mg/dL)	54.75 ± 17.14	48.71 ± 11.82
Triglyceride (mg/dL)	136.56 ± 52.45	117.30 ± 66.61
Albumin (mg/dL)	4.72 ± 0.24	3.79 ± 0.41
Hb (g/dL)	14.34 ± 1.48	12.84 ± 1.27
Hct (%)	42.27 ± 3.88	38.95 ± 4.21
Folate (ng/mL)	10.47 ± 5.20	16.43 ± 11.02
Vitamin B12 (*μ*g/L)	665.10 ± 125.69	824.21 ± 229.73

^∗^Significant difference at *P* < 0.05.

**Table 2:** Frequency distribution of haplotypes and alleles of APOE.

	Control group (*N* = 183)	AD group (*N* = 136)
APOE haplotypes		
*ε*2/*ε*2	2 (1.1%)	—
*ε*2/*ε*3	31 (16.9%)	10 (7.4%)
*ε*3/*ε*3	111 (60.7%)	64 (47.1%)
*ε*2/*ε*4	9 (4.9%)	4 (2.9%)
*ε*3/*ε*4	28 (15.3%)	44 (32.4%)^a^
*ε*4/*ε*4	2 (1.1%)	14 (10.3%)^a^
Allele frequencies		
*ε*2	0.12	0.07
*ε*3	0.77	0.47^a^
*ε*4	0.11	0.46^a^

^a^ *P* value < 0.01 compared between controls and AD groups.

**Table 3:** Upregulated glycoproteins in the serum of the AD group (identified proteins from the UniProt *Homo sapiens* database).

Protein ID	Protein name	Mass	Glycosylation	Posttranslational modification
Q9UP79	A disintegrin and metalloproteinase with thrombospondin motifs 8 (ADAM-TS 8) (ADAM-TS8) (ADAMTS-8) (EC 3.4.24.-) (METH-2) (METH-8)	96,460	N-linked (GlcNAc) asparagine	PTM: the precursor is cleaved by a furin endopeptidase
B7ZC32	Kinesin-like protein KIF28P (kinesin-like protein 6)	108,254		
Q9Y3C8	Ubiquitin-fold modifier-conjugating enzyme 1 (Ufm1-conjugating enzyme 1)	19,458		
P00742	Coagulation factor X (EC 3.4.21.6) (Stuart factor) (Stuart-Prower factor) (cleaved into factor X light chain, factor X heavy chain, and activated factor Xa heavy chain)	54,732	O-linked (GalNAc) threonine	PTM: the vitamin K-dependent, enzymatic carboxylation of some glutamate residues allows the modified protein to bind to calcium
P20340	Ras-related protein Rab-6A (Rab-6)	23,593		PTM: prenylated
Q9NRR8	CDC42 small effector protein 1 (CDC42-binding protein SCIP1) (small effector of CDC42 protein 1)	8,925		
Q9NYH9	U3 small nucleolar RNA-associated protein 6 homolog (hepatocellular carcinoma-associated antigen 66) (multiple hat domain protein)	70,194		
P18077	60S ribosomal protein L35a (cell growth-inhibiting gene 33 protein) (large ribosomal subunit protein eL33)	12,538		
Q3SXY7	Leucine-rich repeat, immunoglobulin-like domain, and transmembrane domain-containing protein 3	74,754	N-linked (GlcNAc) asparagine	PTM: glycosylated
Q9NUL5	Shiftless antiviral inhibitor of ribosomal frameshifting protein (SFL) (SHFL) (interferon-regulated antiviral protein) (IRAV) (repressor of yield of DENV protein) (RyDEN)	33,110		
Q12788	Transducin beta-like protein 3 (WD repeat-containing protein SAZD)	89,035		
O94854	Uncharacterized protein KIAA0754	135,148		
Q9BXJ7	Protein amnionless (cleaved into soluble protein amnionless)	47,754	N-linked (GlcNAc) asparagine	PTM: N-glycosylated
Q09019	Dystrophia myotonica WD repeat-containing protein (Dystrophia myotonica-containing WD repeat motif protein) (protein 59) (protein DMR-N9)	70,438		
Q02846	Retinal guanylyl cyclase 1 (RETGC-1) (EC 4.6.1.2) (CG-E) (guanylate cyclase 2D, retinal) (rod outer segment membrane guanylate cyclase) (ROS-GC)	120,059	N-linked (GlcNAc) asparagine	
Q3LHN2	Keratin-associated protein 19-2	5,737		
O95925	Eppin (cancer/testis antigen 71) (CT71) (epididymal protease inhibitor) (protease inhibitor WAP7) (serine protease inhibitor-like with Kunitz and WAP domain 1) (WAP four-disulfide core domain protein 7)	15,284		
Q9NYW3	Taste receptor type 2 member 7 (T2R7) (taste receptor family B member 4) (TRB4)	36,550	N-linked (GlcNAc) asparagine	
Q6UWV2	Myelin protein zero-like protein 3	25,989	N-linked (GlcNAc) asparagine	
Q9Y5X4	Photoreceptor-specific nuclear receptor (nuclear receptor subfamily 2 group E member 3) (retina-specific nuclear receptor)	44,692		
Q6Y288	Beta-1,3-glucosyltransferase (Beta3Glc-T) (EC 2.4.1.-) (beta 3-glucosyltransferase) (beta-3-glycosyltransferase-like)	56,564	N-linked (GlcNAc) asparagine	
Q8TB45	DEP domain-containing mTOR-interacting protein (DEP domain-containing protein 6)	46,294		PTM: phosphorylated. Phosphorylation weakens interaction with MTOR within mTORC1 and mTORC2
Q9UPG8	Zinc finger protein PLAGL2 (pleiomorphic adenoma-like protein 2)	54,584		
Q9HCG1	Zinc finger protein 160 (zinc finger protein HZF5) (zinc finger protein Kr18) (HKr18)	94,112		
P30281	G1/S-specific cyclin-D3	32,520		PTM: polyubiquitinated by the SCF(FBXL2) complex, leading to proteasomal degradation
A0A286YF01	Small cysteine and glycine repeat-containing protein 7 (keratin-associated protein 28-7)	8,951		
O15539	Regulator of G protein signaling 5 (RGS5)	20,946		
Q12979	Active breakpoint cluster region-related protein	97,598		
Q96EN8	Molybdenum cofactor sulfurase (MCS) (MOS) (MoCo sulfurase) (hMCS) (EC 2.8.1.9) (molybdenum cofactor sulfurtransferase)	98,120		
P13765	HLA class II histocompatibility antigen, DO beta chain (MHC class II antigen DOB)	30,822	N-linked (GlcNAc asparagine	
Q6L8H2	Keratin-associated protein 5-3 (keratin-associated protein 5-9) (keratin-associated protein 5.3) (keratin-associated protein 5.9) (UHS KerB-like) (ultrahigh sulfur keratin-associated protein 5.3)	22,106		
P21941	Cartilage matrix protein (Matrilin-1)	53,701	N-linked (GlcNAc) asparagine	
Q6IFN5	Olfactory receptor 7E24 (olfactory receptor OR19-14)	38,279	N-linked (GlcNAc) asparagine	
Q96BH3	Epididymal sperm-binding protein 1 (epididymal secretory protein 12) (hE12)	26,106	N-linked (GlcNAc) asparagine	PTM: N-glycosylated
O95149	Snurportin-1 (RNA U transporter 1)	41,143		
Q9BSJ5	Uncharacterized protein C17orf80 (cell migration-inducing gene 3 protein) (human lung cancer oncogene 8 protein) (HLC-8)	67,315		
Q9H1E5	Thioredoxin-related transmembrane protein 4 (thioredoxin domain-containing protein 13)	38,952		
Q8NCL4	Polypeptide N-acetylgalactosaminyltransferase 6 (EC 2.4.1.41) (polypeptide GalNAc transferase 6) (GalNAc-T6) (pp-GaNTase 6) (protein-UDP acetylgalactosaminyltransferase 6) (UDP-GalNAc:polypeptide N-acetylgalacto saminyltransferase 6)	71,159	N-linked (GlcNAc) asparagine	
Q4G112	Heat shock factor protein 5 (HSF 5) (heat shock transcription factor 5) (HSTF 5)	65,278		
Q9Y6L6	Solute carrier organic anion transporter family member 1B1 (liver-specific organic anion transporter 1) (LST-1) (OATP-C) (sodium-independent organic anion-transporting polypeptide 2) (OATP-2) (solute carrier family 21 member 6)	76,449	N-linked (GlcNAc) asparagine	
P55073	Thyroxine 5-deiodinase (EC 1.21.99.3) (5DIII) (DIOIII) (type 3 DI) (type III iodothyronine deiodinase)	33,947		
Q9H694	Protein bicaudal C homolog 1 (Bic-C)	104,844		

**Table 4:** Downregulated glycoproteins in the serum of the AD group (identified proteins from the UniProt *Homo sapiens* database).

Protein ID	Protein names	Mass	Glycosylation	Posttranslational modification
Q9UBG0	C-type mannose receptor 2 (C-type lectin domain family 13 member E) (endocytic receptor 180) (macrophage mannose receptor 2) (urokinase-type plasminogen activator receptor-associated protein) (UPAR-associated protein) (urokinase receptor-associated protein) (CD antigen CD280)	166,674	N-linked (GlcNAc) (complex) asparagine	PTM: N-glycosylated
Q96CS3	FAS-associated factor 2 (protein ETEA) (UBX domain-containing protein 3B) (UBX domain-containing protein 8)	52,623		
Q9Y6E7	NAD-dependent protein lipoamidase sirtuin-4, mitochondrial (EC 2.3.1.-) (NAD-dependent ADP-ribosyltransferase sirtuin-4) (EC 2.4.2.-) (NAD-dependent protein deacetylase sirtuin-4) (EC 2.3.1.286) (regulatory protein SIR2 homolog 4) (SIR2-like protein 4)	35,188		
Q8N6S4	Ankyrin repeat domain-containing protein 13C	60,818		
Q9UHR4	Brain-specific angiogenesis inhibitor 1-associated protein 2-like protein 1 (BAI1-associated protein 2-like protein 1) (insulin receptor tyrosine kinase substrate)	56,883		PTM: Phosphorylated on tyrosine in response to insulin
B2RUY7	von Willebrand factor C domain-containing protein 2-like (Brorin-like)	24,570		
Q9Y3F4	Serine-threonine kinase receptor-associated protein (MAP activator with WD repeats) (UNR-interacting protein) (WD-40 repeat protein PT-WD)	38,438		
Q8TCV5	WAP four-disulfide core domain protein 5 (putative protease inhibitor WAP1) (p53-responsive gene 5 protein)	24,238		
O60568	Multifunctional procollagen lysine hydroxylase and glycosyltransferase LH3 (including procollagen-lysine, 2-oxoglutarate 5-dioxygenase 3 (EC 1.14.11.4) (lysyl hydroxylase 3) (LH3); procollagen glycosyltransferase (EC 2.4.1.50) (EC 2.4.1.66) (galactosylhydroxylysine-glucosyltransferase) (procollagen galactosyltransferase)	84,785	N-linked (GlcNAc) asparagine	
Q7Z7J5	Developmental pluripotency-associated protein 2 (pluripotent embryonic stem cell-related gene 1 protein)	33,784		
Q8N6C5	Immunoglobulin superfamily member 1 (IgSF1) (immunoglobulin-like domain-containing protein 1) (inhibin-binding protein) (InhBP) (pituitary gland-specific factor 2) (p120)	148,936	N-linked (GlcNAc) asparagine	
Q04837	Single-stranded DNA-binding protein, mitochondrial (Mt-SSB) (MtSSB) (PWP1-interacting protein 17)	17,260		
Q7RTX1	Taste receptor type 1 member 1 (G protein-coupled receptor 70)	93,074	N-linked (GlcNAc) asparagine	
P13994	Coiled-coil domain-containing protein 130 (9 kDa protein)	44,802		
Q5T440	Putative transferase CAF17, mitochondrial (EC 2.1.-.-) (iron-sulfur cluster assembly factor homolog)	38,155		
A7E2S9	Putative ankyrin repeat domain-containing protein 30B-like	28,549		
Q6QNK2	Adhesion G protein-coupled receptor D1 (G protein-coupled receptor 133) (G protein-coupled receptor PGR25)	96,530	N-linked (GlcNAc) asparagine	
Q8NBR0	Tumor protein p53-inducible protein 13 (damage-stimulated cytoplasmic protein 1)	42,238		
A2VDJ0	Transmembrane protein 131-like	179,339	N-linked (GlcNAc) asparagine	
Q9NZT2	Opioid growth factor receptor (OGFr) (protein 7-60) (zeta-type opioid receptor)	73,325		
P62910	60S ribosomal protein L32 (large ribosomal subunit protein eL32)	15,860		
Q8WXI2	Connector enhancer of kinase suppressor of ras 2 (connector enhancer of KSR 2) (CNK homolog protein 2) (CNK2)	117,535		PTM: phosphorylated on tyrosine
O95396	Adenylyltransferase and sulfurtransferase MOCS3 (molybdenum cofactor synthesis protein 3) (molybdopterin synthase sulfurylase) (MPT synthase sulfurylase) (including molybdopterin-synthase adenylyltransferase (EC 2.7.7.80) (adenylyltransferase MOCS3) (sulfur carrier protein MOCS2A adenylyltransferase); molybdopterin-synthase sulfurtransferase (EC 2.8.1.11) (sulfur carrier protein MOCS2A sulfurtransferase) (sulfurtransferase MOCS3))	49,669		
Q8IVN8	Somatomedin-B and thrombospondin type-1 domain-containing protein (RPE-spondin)	29,610	N-linked (GlcNAc) asparagine	
P55055	Oxysterol receptor LXR-beta (liver X receptor beta) (nuclear receptor NER) (nuclear receptor subfamily 1 group H member 2) (ubiquitously expressed nuclear receptor)	50,974		PTM: sumoylated by SUMO2 at Lys-409 and Lys-447 during the hepatic acute phase response
Q7Z3I7	Zinc finger protein 572	61,238		
Q9Y580	RNA-binding protein 7 (RNA-binding motif protein 7)	30,504		PTM: phosphorylated at Ser-136 by MAPK14/p38-alpha-activated MAPKAPK2/MK2
P48729	Casein kinase I isoform alpha (CKI-alpha) (EC 2.7.11.1) (CK1)	38,915		
P19532	Transcription factor E3 (class E basic helix-loop-helix protein 33) (bHLHe33)	61,521		PTM: phosphorylation by MTOR regulates its subcellular location and activity
Q8WXH4	Ankyrin repeat and SOCS box protein 11 (ASB-11)	35,367		
Q86U42	Polyadenylate-binding protein 2 (PABP-2) (poly(A)-binding protein 2) (nuclear poly(A)-binding protein 1) (poly(A)-binding protein II) (PABII) (polyadenylate-binding nuclear protein 1)	32,749		PTM: arginine dimethylation is asymmetric and involves PRMT1 and PRMT3
Q96MT1	RING finger protein 145 (EC 2.3.2.27)	75,617		
P17643	5,6-Dihydroxyindole-2-carboxylic acid oxidase (DHICA oxidase) (EC 1.14.18.-) (catalase B) (glycoprotein 75) (melanoma antigen gp75) (tyrosinase-related protein 1) (TRP) (TRP-1) (TRP1)	60,724	N-linked (GlcNAc) asparagine	PTM: glycosylated
Q9NW75	G patch domain-containing protein 2	58,944		
Q09472	Histone acetyltransferase p300 (p300 HAT) (EC 2.3.1.48) (E1A-associated protein p300) (histone butyryltransferase p300) (EC 2.3.1.-) (histone crotonyltransferase p300) (EC 2.3.1.-) (protein 2-hydroxyisobutyryltransferase p300) (EC 2.3.1.-) (protein lactyltransferase p300) (EC 2.3.1.-) (protein propionyltransferase p300) (EC 2.3.1.-)	264,161		PTM: acetylated on Lys at up to 17 positions by intermolecular autocatalysis
P58173	Olfactory receptor 2B6 (Hs6M1-32) (olfactory receptor 2B1) (olfactory receptor 2B5) (olfactory receptor 5-40) (OR5-40) (olfactory receptor 6-31) (OR6-31) (olfactory receptor OR6-4)	35,414	N-linked (GlcNAc) asparagine	
Q15776	Zinc finger protein with KRAB and SCAN domains 8 (LD5-1) (zinc finger protein 192)	65,816		

**Table 5:** Glycoprotein classification in AD patients: upregulated and downregulated.

Classification	Percentage
Upregulated glycoproteins	
Protein-binding activity modulator (PC00095)	16.70%
Gene-specific transcriptional regulator (PC00264)	12.50%
Metabolite interconversion enzyme (PC00262)	12.50%
Protein-modifying enzyme (PC00260)	8.30%
Transporter (PC00227)	8.30%
Nucleic acid metabolism protein (PC00171)	8.30%
Transmembrane signal receptor (PC00197)	8.30%
Scaffold/adaptor protein (PC00226)	4.20%
Defense/immunity protein (PC00090)	4.20%
Cytoskeletal protein (PC00085)	4.20%
Structural protein (PC00211)	4.20%
Extracellular matrix protein (PC00102)	4.20%
Translational protein (PC00263)	4.20%
Downregulated glycoproteins	
Metabolite interconversion enzyme (PC00262)	13.70%
Nucleic acid metabolism protein (PC00171)	12.40%
Protein-modifying enzyme (PC00260)	12.40%
Gene-specific transcriptional regulator (PC00264)	9.90%
Transmembrane signal receptor (PC00197)	9.30%
Scaffold/adaptor protein (PC00226)	6.80%
Transporter (PC00227)	6.20%
Protein-binding activity modulator (PC00095)	5.00%
Translational protein (PC00263)	5.00%
Defense/immunity protein (PC00090)	3.70%
Intercellular signal molecule (PC00207)	3.10%
Cytoskeletal protein (PC00085)	2.50%
Chromatin/chromatin-binding or chromatin-regulatory protein (PC00077)	2.50%
Membrane traffic protein (PC00150)	2.50%
Cell adhesion molecule (PC00069)	1.90%
Structural protein (PC00211)	1.20%
Extracellular matrix protein (PC00102)	0.60%
Chaperone (PC00072)	0.60%
Cell junction protein (PC00070)	0.60%

**Table 6:** Unique glycoproteins in the AD group classified by the molecular function, biological process, and pathways.

Classification	Percentage (%)	Classification	Percentage (%)
Molecular function		Pathways	
Organic cyclic compound binding (GO:0097159)	26.0	Alzheimer disease-presenilin pathway (P00004)	16.60%
Heterocyclic compound binding (GO:1901363)	26.0	Inflammation mediated by chemokine and cytokine signaling pathway (P00031)	9.20%
Protein binding (GO:0005515)	24.7	Apoptosis signaling pathway (P00006)	5.10%
Ion binding (GO:0043167)	5.5	Integrin signaling pathway (P00034)	5.10%
Small molecule binding (GO:0036094)	5.5	Wnt signaling pathway (P00057)	5.10%
Carbohydrate binding (GO:0030246)	5.6	p53 pathway (P00059)	3.70%
Carbohydrate derivative binding (GO:0097367)	4.1	Metabotropic glutamate receptor group III pathway (P00039)	2.60%
Protein-containing complex binding (GO:0044877)	2.7	Ionotropic glutamate receptor pathway (P00037)	2.60%
Biological process		Gamma-aminobutyric acid synthesis (P04384)	2.60%
Cellular process (GO:0009987)	32.30%	Dopamine receptor-mediated signaling pathway (P05912)	2.60%
Metabolic process (GO:0008152)	15.50%	p53 pathway feedback loops 2 (P04398)	2.60%
Biological regulation (GO:0065007)	15.10%	Endothelin signaling pathway (P00019)	2.60%
Response to stimulus (GO:0050896)	9.20%	Parkinson disease (P00049)	2.60%
Localization (GO:0051179)	8.00%	EGF receptor signaling pathway (P00018)	2.60%
Signaling (GO:0023052)	6.40%	PDGF signaling pathway (P00047)	2.60%
Developmental process (GO:0032502)	4.80%	Ras pathway (P04393)	2.60%
Multicellular organismal process (GO:0032501)	4.80%	Notch signaling pathway (P00045)	2.60%
Others	4.0%	Muscarinic acetylcholine receptor 2 and 4 signaling pathway (P00043)	2.60%
		Cadherin signaling pathway (P00012)	2.60%
		Metabotropic glutamate receptor group I pathway (P00041)	2.60%
Carbohydrate derivative binding (GO:0097367)	4.1	Metabotropic glutamate receptor group II pathway (P00040)	2.60%
		CCKR signaling map (P06959)	2.60%
		Beta2 adrenergic receptor signaling pathway (P04378)	2.60%
		Formyltetrahydroformate biosynthesis (P02743)	2.60%
		Beta1 adrenergic receptor signaling pathway (P04377)	2.60%
		5HT1 type receptor-mediated signaling pathway (P04373)	2.60%
		Transcription regulation by bZIP transcription factor (P00055)	2.60%
		GABA-B receptor II signaling (P05731)	2.60%
		Enkephalin release (P05913)	1.50%
		B cell activation (P00010)	1.00%

## Data Availability

The data supporting these findings are available from the corresponding author upon reasonable request.

## Abbreviations

A*β*: Amyloid beta
AD: Alzheimer's disease
ADAM10: A disintegrin and metalloproteinase
ADAMTS8: A disintegrin and metalloproteinase with thrombospondin motifs 8
AGEs: Advanced glycation end products
ANKRD13C: Ankyrin repeat domain-containing protein 13C
APOE: Apolipoprotein E
APP: Amyloid-beta precursor protein
ARHGEF12: Rho guanine nucleotide exchange factor 12
ATP13A2: Polyamine-transporting ATPase 13A2
BAIAP2L1: Brain-specific angiogenesis inhibitor 1-associated protein 2-like protein 1
CASP10: Caspase 10
CIT: Citron Rho-interacting kinase
EC: Endothelial cell
ECLIA: Electrochemiluminescence immunoassays
EOAD: Early-onset AD
DSP: Desmoplakin
FADD: FAS-associated death domain protein
FAF2: FAS-associated factor 2
FBG: Fasting blood glucose
FX: Coagulation factor X
GlcNAC: N-Acetyl glucosamine
GPCRs: G protein-coupled receptors
GSK3*β*: Glycogen synthase kinase 3*β*
HbA1c: Glycated hemoglobin
HDL-cholesterol: High-density lipoprotein cholesterol
MAPT: Microtubule-associated protein tau
MCP-1: Macrophage chemotactic protein-1
MRC2: C-type mannose receptor 2
NRG2: Proneuregulin-2, membrane-bound isoform
IL: Interleukin
IRTKS: Insulin receptor tyrosine kinase substrate
LC/LC-MS/MS: Liquid chromatography and tandem mass spectrometry
LDL-cholesterol: Low-density lipoprotein cholesterol
LOAD: Late-onset AD
LRP1: LDL receptor-related protein
KEGG: Kyoto Encyclopedia of Genes and Genomes
KIF28P: Kinesin-like protein KIF28P
PCR: Polymerase chain reaction
PLD3: Phospholipase D3
PDZRN3: PDZ domain-containing RING finger protein 3
PHLP3: Phosducin-like protein 3
PI3K: Phosphatidylinositol-3-kinase
PKB/Akt: Protein kinase B
PPP1R9A: Protein phosphatase 1 regulatory subunit 9A
PSEN1: Presenilin 1
PSEN2: Presenilin 2
RAB6A: Ras-related protein Rab-6A
RAGEs: Receptor of advanced glycation end products
SIRT4: NAD-dependent protein lipoamidase sirtuin-4, mitochondrial
TG: Triglyceride
TNF: Tumor necrosis factor
TXNDC9: Thioredoxin domain-containing protein 9
Wnt: Wingless-type MMTV integration site family member
UFC1: Ubiquitin-fold modifier-conjugating enzyme 1.

## Supplementary Materials

Supplementary Materials Data in this part is presented in Supplementary Table 1 that identifies all downregulated glycoproteins from serum of the AD group.

## References

[1] Alzheimer's Association Report (2021). 2021 Alzheimer's disease facts and figures. *Alzheimer's & Dementia*.

[2] He W., Goodkind D., Kowal P., US Census Bureau, International Population Reports, P95/16-1 (2016). *An Aging World: 2015*.

[3] Rajan K. B., Weuve J., Barnes L. L., McAninch E. A., Wilson R. S., Evans D. A. (2021). Population estimate of people with clinical AD and mild cognitive impairment in the United States (2020–2060).

[4] Cui L., Hou N. N., Wu H. M. (2020). Prevalence of Alzheimer's disease and Parkinson's disease in China: an updated systematical analysis. *Frontiers in Aging Neuroscience*.

[5] Wangtongkum S., Sucharitkul P., Silprasert N., Inthrachak R. (2008). Prevalence of dementia among population age over 45 years in Chiang Mai, Thailand. *Journal of the Medical Association of Thailand*.

[6] LaFerla F. M., Green K. N. (2012). Animal models of Alzheimer disease. *Cold Spring Harbor Perspectives in Medicine*.

[7] Mehlhorn G., Hollborn M., Schliebs R. (2000). Induction of cytokines in glial cells surrounding cortical beta-amyloid plaques in transgenic Tg2576 mice with Alzheimer pathology. *International Journal of Developmental Neuroscience*.

[8] Lue L. F., Sabbagh M. N., Chiu M. J. (2017). Plasma levels of A*β*42 and tau identified probable Alzheimer's dementia: findings in two cohorts. *Frontiers in Aging Neuroscience*.

[9] Butterfield D. A., Castegna A. (2003). Proteomics for the identification of specifically oxidized proteins in brain: technology and application to the study of neurodegenerative disorders. *Amino Acids*.

[10] Anderson N. L., Anderson N. G. (2002). The Human Plasma Proteome:. *Molecular & Cellular Proteomics*.

[11] Dey K. K., Wang H., Niu M. (2019). Deep undepleted human serum proteome profiling toward biomarker discovery for Alzheimer's disease. *Clinical Proteomics*.

[12] Frost D. C., Li L. (2014). Recent advances in mass spectrometry-based glycoproteomics. *Advances in Protein Chemistry and Structural Biology*.

[13] Pan S., Chen R., Aebersold R., Brentnall T. A. (2011). Mass Spectrometry Based Glycoproteomics--From a Proteomics Perspective. *Molecular & Cellular Proteomics*.

[14] Zhang Q., Ma C., Chin L. S., Lian L. (2020). Integrative glycoproteomics reveals protein N-glycosylation aberrations and glycoproteomic network alterations in Alzheimer's disease. *Science Advances*.

[15] McKhann G. M., Knopman D. S., Chertkow H. (2011). The diagnosis of dementia due to Alzheimer's disease: recommendations from the National Institute on Aging-Alzheimer's Association workgroups on diagnostic guidelines for Alzheimer's disease. *Alzheimer's & Dementia*.

[16] Lowry O., Rosebrough N., Farr A. L., Randall R. J. (1951). Protein measurement with the folin phenol reagent. *The Journal of Biological Chemistry*.

[17] Bardou P., Mariette J., Escudié F., Djemiel C., Klopp C. (2014). Jvenn: an interactive Venn diagram viewer. *BMC Bioinformatics*.

[18] Nixon R. A., Cataldo A. M., Mathews P. M. (2000). The endosomal-lysosomal system of neurons in Alzheimer's disease pathogenesis: a review. *Neurochemical Research*.

[19] Caccamo A., Magrì A., Medina D. X. (2013). mTOR regulates tau phosphorylation and degradation: implications for Alzheimer's disease and other tauopathies. *Aging Cell*.

[20] Caamaño-Isorna F., Corral M., Montes-Martínez A., Takkouche B. (2006). Education and dementia: a meta-analytic study. *Neuroepidemiology*.

[21] Qiu C., Bäckman L., Winblad B., Agüero-Torres H., Fratiglioni L. (2001). The influence of education on clinically diagnosed dementia incidence and mortality data from the Kungsholmen Project. *Archives of Neurology*.

[22] Sims R., Hill M., Williams J. (2020). The multiplex model of the genetics of Alzheimer's disease. *Nature Neuroscience*.

[23] Crean S., Ward A., Mercaldi C. J. (2011). Apolipoprotein E *ε*4 prevalence in Alzheimer's disease patients varies across global populations: a systematic literature review and meta-analysis. *Dementia and Geriatric Cognitive Disorders*.

[24] Oliveira F. F., Chen E. S., Smith M. C., Bertolucci P. H. F. (2016). Predictors of cognitive and functional decline in patients with Alzheimer disease dementia from Brazil. *Alzheimer Disease and Associated Disorders*.

[25] Yamazaki Y., Zhao N., Caulfield T. R., Liu C.-C., Bu G. (2019). Apolipoprotein E and Alzheimer disease: pathobiology and targeting strategies. *Nature Reviews. Neurology*.

[26] Hondius D. C., van Nierop P., Li K. W. (2016). Profiling the human hippocampal proteome at all pathologic stages of Alzheimer's disease. *Alzheimer's & Dementia*.

[27] Sathe G., Na C. H., Renuse S. (2019). Quantitative proteomic profiling of cerebrospinal fluid to identify candidate biomarkers for Alzheimer's disease. *Proteomics. Clinical Applications*.

[28] Gurses M. S., Ural M. N., Gulec M. A., Akyol O., Akyol S. (2016). Pathophysiological function of ADAMTS enzymes on molecular mechanism of Alzheimer's disease. *Aging and Disease*.

[29] Yu N.-N., Tan M.-S., Yu J.-T., Xie A.-M., Tan L. (2016). The role of Reelin signaling in Alzheimer's disease. *Molecular Neurobiology*.

[30] Krstic D., Rodriguez M., Knuesel I. (2012). Regulated proteolytic processing of Reelin through interplay of tissue plasminogen activator (tPA), ADAMTS-4, ADAMTS-5, and their modulators. *PLoS One*.

[31] Hisanaga A., Morishita S., Suzuki K. (2012). A disintegrin and metalloproteinase with thrombospondin motifs 4 (ADAMTS-4) cleaves Reelin in an isoform-dependent manner. *FEBS Letters*.

[32] Gerakis Y., Quintero M., Li H., Hetz C. (2019). The UFMylation system in proteostasis and beyond. *Trends in Cell Biology*.

[33] Gerakis Y., Hetz C. (2018). Emerging roles of ER stress in the etiology and pathogenesis of Alzheimer's disease. *The FEBS Journal*.

[34] Hung C. O., Coleman M. P. (2016). KIF1A mediates axonal transport of BACE1 and identification of independently moving cargoes in living SCG neurons. *Traffic*.

[35] Kandalepas P. C., Sadleir K. R., Eimer W. A., Zhao J., Nicholson D. A., Vassar R. (2013). The Alzheimer’s *β*-secretase BACE1 localizes to normal presynaptic terminals and to dystrophic presynaptic terminals surrounding amyloid plaques. *Acta Neuropathologica*.

[36] Zhao J., Fu Y., Yasvoina M. (2007). Site Amyloid Precursor Protein Cleaving Enzyme 1 Levels Become Elevated in Neurons around Amyloid Plaques: Implications for Alzheimer's Disease Pathogenesis. *The Journal of Neuroscience*.

[37] Zhang X., Huang T. Y., Yancey J., Luo H., Zhang Y.-W. (2019). Role of Rab GTPases in Alzheimer's disease. *ACS Chemical Neuroscience*.

[38] Scheper W., Hoozemans J. J., Hoogenraad C. C., Rozemuller A. J. M., Eikelenboom P., Baas F. (2007). Rab6 is increased in Alzheimer’s disease brain and correlates with endoplasmic reticulum stress. *Neuropathology and Applied Neurobiology*.

[39] McConlogue L., Castellano F., deWit C., Schenk D., Maltese W. A. (1996). Differential Effects of a Rab6 Mutant on Secretory _Versus_ Amyloidogenic Processing of Alzheimer's *β*-Amyloid Precursor Protein. *The Journal of Biological Chemistry*.

[40] Venkateswarlu D., Perera L., Darden T., Pedersen L. G. (2002). Structure and dynamics of zymogen human blood coagulation factor X. *Biophysical Journal*.

[41] Grammas P. (2011). Neurovascular dysfunction, inflammation and endothelial activation: implications for the pathogenesis of Alzheimer's disease. *Journal of Neuroinflammation*.

[42] Lee D. Y., Park K. W., Jin B. K. (2006). Thrombin induces neurodegeneration and microglial activation in the cortex _in vivo_ and _in vitro_ : Proteolytic and non-proteolytic actions. *Biochemical and Biophysical Research Communications*.

[43] Jürgensen H. J., Johansson K., Madsen D. H. (2014). Complex Determinants in Specific Members of the Mannose Receptor Family Govern Collagen Endocytosis. *The Journal of Biological Chemistry*.

[44] Cataldo A. M., Peterhoff C. M., Troncoso J. C., Gomez-Isla T., Hyman B. T., Nixon R. A. (2000). Endocytic Pathway Abnormalities Precede Amyloid *β* Deposition in Sporadic Alzheimer's Disease and Down Syndrome: Differential Effects of APOE Genotype and Presenilin Mutations. *The American Journal of Pathology*.

[45] Galatro T. F., Holtman I. R., Lerario A. M. (2017). Transcriptomic analysis of purified human cortical microglia reveals age- associated changes. *Nature Neuroscience*.

[46] Stevenson J., Huang E. Y., Olzmann J. A. (2016). Endoplasmic reticulum-associated degradation and lipid homeostasis. *Annual Review of Nutrition*.

[47] P Bennett J., M Keeney P. (2017). Micro RNA’s (mirna’s) may help explain expression of multiple genes in Alzheimer’s Frontal Cortex. *Journal of Systems and Integrative Neuroscience*.

[48] Julien C., Tremblay C., Émond V. (2009). Sirtuin 1 reduction parallels the accumulation of tau in Alzheimer disease. *Journal of Neuropathology and Experimental Neurology*.

[49] Cieślik M., Czapski G. A., Strosznajder J. B. (2015). The molecular mechanism of amyloid *β*42 peptide toxicity: the role of sphingosine kinase-1 and mitochondrial sirtuins. *PLoS One*.

[50] Parent A., Roy S. J., Iorio-Morin C. (2010). ANKRD13C Acts as a Molecular Chaperone for G Protein-coupled Receptors. *The Journal of Biological Chemistry*.

[51] Zhao J., Deng Y., Jiang Z., Qing H. (2016). G Protein-coupled receptors (GPCRs) in Alzheimer's disease: a focus on BACE1 related GPCRs. *Frontiers in Aging Neuroscience*.

[52] Wu C., Cui X., Huang L. (2019). IRTKS promotes insulin signaling transduction through inhibiting SHIP2 phosphatase activity. *International Journal of Molecular Sciences*.

[53] Huang L. Y., Wang Y. P., Wei B. F. (2013). Deficiency of IRTKS as an adaptor of insulin receptor leads to insulin resistance. *Cell Research*.

[54] Kellar D., Craft S. (2020). Brain insulin resistance in Alzheimer's disease and related disorders: mechanisms and therapeutic approaches. *Lancet Neurology*.

[55] Chen M., Xia W. (2020). Proteomic profiling of plasma and brain tissue from Alzheimer's disease patients reveals candidate network of plasma biomarkers. *Journal of Alzheimer's Disease*.

[56] Yuan X. Z., Sun S., Tan C. C., Yu J.-T., Tan L. (2017). The role of ADAM10 in Alzheimer's disease. *Journal of Alzheimer's Disease*.

[57] Shinohara M., Tachibana M., Kanekiyo T., Bu G. (2017). Role of LRP1 in the pathogenesis of Alzheimer's disease: evidence from clinical and preclinical studies:. *Journal of Lipid Research*.

[58] van Veen S., Martin S., van den Haute C. (2020). ATP13A2 deficiency disrupts lysosomal polyamine export. *Nature*.

[59] Ledonne A., Mercuri N. B. (2020). On the modulatory roles of neuregulins/ErbB signaling on synaptic plasticity. *International Journal of Molecular Sciences*.

[60] Hermkens D. M. A., Stam O. C. G., de Wit N. M. (2019). Profiling the unique protective properties of intracranial arterial endothelial cells. *Acta Neuropathologica Communications*.

[61] Hye A., Lynham S., Thambisetty M. (2006). Proteome-based plasma biomarkers for Alzheimer’s disease. *Brain*.

[62] Erickson J. W., Cerione R. A. (2004). Structural elements, mechanism, and evolutionary convergence of rho protein-guanine nucleotide exchange factor complexes. *Biochemistry*.

[63] Aguilar B. J., Zhu Y., Lu Q. (2017). Rho GTPases as therapeutic targets in Alzheimer's disease. *Alzheimer's Research & Therapy*.

[64] Borin M., Saraceno C., Catania M. (2018). Rac1 activation links tau hyperphosphorylation and A*β* dysmetabolism in Alzheimer's disease. *Acta Neuropathologica Communications*.

[65] Braithwaite S. P., Stock J. B., Lombroso P. J., Nairn A. C. (2012). Protein phosphatases and Alzheimer's disease. *Progress in Molecular Biology and Translational Science*.

[66] McIlwain D. R., Berger T., Mak T. W. (2013). Caspase functions in cell death and disease. *Cold Spring Harbor Perspectives in Biology*.

[67] Rehker J., Rodhe J., Nesbitt R. R. (2017). Caspase-8, association with Alzheimer's disease and functional analysis of rare variants. *PLoS One*.

[68] Lee E. W., Seo J., Jeong M., Lee S., Song J. (2012). The roles of FADD in extrinsic apoptosis and necroptosis. *BMB Reports*.

[69] Wu C. K., Thal L., Pizzo D., Hansen L., Masliah E., Geula C. (2005). Apoptotic signals within the basal forebrain cholinergic neurons in Alzheimer's disease. *Experimental Neurology*.

[70] Maier O., Fischer R., Agresti C., Pfizenmaier K. (2013). TNF receptor 2 protects oligodendrocyte progenitor cells against oxidative stress. *Biochemical and Biophysical Research Communications*.

[71] Jiang H., He P., Xie J., Staufenbiel M., Li R., Shen Y. (2014). Genetic deletion of TNFRII gene enhances the Alzheimer-like pathology in an APP transgenic mouse model via reduction of phosphorylated I*κ*B*α*. *Human Molecular Genetics*.

[72] Sewduth R., Jaspard-Vinassa B., Peghaire C. (2014). The ubiquitin ligase PDZRN3 is required for vascular morphogenesis through Wnt/planar cell polarity signalling. *Nature Communications*.

[73] Harris L. D., Jasem S., Licchesi J. D. F. (2020). The ubiquitin system in Alzheimer's disease. *Advances in Experimental Medicine and Biology*.

[74] Gavin A. L., Huang D., Huber C. (2018). PLD3 and PLD4 are single-stranded acid exonucleases that regulate endosomal nucleic-acid sensing. *Nature Immunology*.

[75] Scholtzova H., Kascsak R. J., Bates K. A. (2009). Induction of toll-like receptor 9 signaling as a method for ameliorating Alzheimer's disease-related pathology. *The Journal of Neuroscience*.

[76] Stirling P. C., Cuéllar J., Alfaro G. A. (2006). PhLP3 Modulates CCT-mediated Actin and Tubulin Folding via Ternary Complexes with Substrates. *The Journal of Biological Chemistry*.

[77] Schuller E., Gulesserian T., Seidl R., Cairns N., Lubec G. (2001). Brain t-complex polypeptide 1 (TCP-1) related to its natural substrate *β*1 tubulin is decreased in Alzheimer's disease. *Life Sciences*.

[78] Sharp E. S., Gatz M. (2011). Relationship between education and dementia: an updated systematic review. *Alzheimer Disease and Associated Disorders*.

