# Supplementary material for: Serum Glycoproteomics and Identification of Potential Mechanisms Underlying Alzheimer's Disease
Source: Behav Neurol. 2021 Dec 11;2021:1434076. doi: 10.1155/2021/1434076 (PMC8684523; doi:10.1155/2021/1434076)
Supplement: Supplementary Materials — Data in this part is presented in Supplementary Table 1 that identifies all downregulated glycoproteins from serum of the AD group. [file 1434076.f1.pdf]

Supplementary Table 1. Down-regulated glycoproteinsin serum AD group (Identified proteins from Uniprot *homo sapiens* database)

Protein ID	Protein names	Mass	Glycosylation	Post-translational modification
Q9UBG0	C-type mannose receptor 2 (C-type lectin domain family 13 member E) (Endocytic receptor 180) (Macrophage mannose receptor 2) (Urokinase-type plasminogen activator receptor-associated protein) (UPAR-associated protein) (Urokinase receptor-associated protein) (CD antigen CD280)	166,674	N-linked (GlcNAc...) (complex) asparagine	PTM: N-glycosylated.
Q96CS3	FAS-associated factor 2 (Protein ETEA) (UBX domain-containing protein 3B) (UBX domain-containing protein 8)	52,623		
Q9Y6E7	NAD-dependent protein lipoamidase sirtuin-4, mitochondrial (EC 2.3.1.-) (NAD-dependent ADP-ribosyltransferase sirtuin-4) (EC 2.4.2.-) (NAD-dependent protein deacetylase sirtuin-4) (EC 2.3.1.286) (Regulatory protein SIR2 homolog 4) (SIR2-like protein 4)	35,188		
Q8N6S4	Ankyrin repeat domain-containing protein 13C	60,818		
Q9UHR4	Brain-specific angiogenesis inhibitor 1-associated protein 2-like protein 1 (BAI1-associated protein 2-like protein 1) (Insulin receptor tyrosine kinase substrate)	56,883		PTM: Phosphorylated on tyrosine in response to insulin
B2RUY7	von Willebrand factor C domain-containing protein 2-like (Brorin-like)	24,570		
Q9Y3F4	Serine-threonine kinase receptor-associated protein (MAP activator with WD repeats) (UNR-interacting protein) (WD-40 repeat protein PT-WD)	38,438		
Q8TCV5	WAP four-disulfide core domain protein 5 (Putative protease inhibitor WAP1) (p53-responsive gene 5 protein)	24,238		
O60568	Multifunctional procollagen lysine hydroxylase and glycosyltransferase LH3 [Includes: Procollagen-lysine,2-oxoglutarate 5-dioxygenase 3 (EC 1.14.11.4) (Lysyl hydroxylase 3) (LH3); Procollagen glycosyltransferase (EC 2.4.1.50) (EC 2.4.1.66) (Galactosylhydroxylysine-glucosyltransferase) (Procollagen galactosyltransferase)	84,785	N-linked (GlcNAc...) asparagine	
Q7Z7J5	Developmental pluripotency-associated protein 2 (Pluripotent embryonic stem cell-related gene 1 protein)	33,784		
Q8N6C5	Immunoglobulin superfamily member 1 (IgSF1) (Immunoglobulin-like domain-containing protein 1) (Inhibin-binding protein) (InhBP) (Pituitary gland-specific factor 2) (p120)	148,936	N-linked (GlcNAc...) asparagine	
Q04837	Single-stranded DNA-binding protein, mitochondrial (Mt-SSB) (MtSSB) (PWP1-interacting protein 17)	17,260		
Q7RTX1	Taste receptor type 1 member 1 (G-protein coupled receptor 70)	93,074	N-linked (GlcNAc...) asparagine	
P13994	Coiled-coil domain-containing protein 130 (9 kDa protein)	44,802		
Q5T440	Putative transferase CAF17, mitochondrial (EC 2.1.-.-) (Iron-sulfur cluster assembly factor homolog)	38,155		
A7E2S9	Putative ankyrin repeat domain-containing protein 30B-like	28,549		
Q6QNK2	Adhesion G-protein coupled receptor D1 (G-protein coupled receptor 133) (G-protein coupled receptor PGR25)	96,530	N-linked (GlcNAc...) asparagine	
Q8NBR0	Tumor protein p53-inducible protein 13 (Damage-stimulated cytoplasmic protein 1)	42,238		
A2VDJ0	Transmembrane protein 131-like	179,339	N-linked (GlcNAc...) asparagine	
Q9NZT2	Opioid growth factor receptor (OGFr) (Protein 7-60) (Zeta-type opioid receptor	73,325		

Protein ID	Protein names	Mass	Glycosylation	Post-translational modification
P62910	60S ribosomal protein L32 (Large ribosomal subunit protein eL32)	15,860		
Q8WXI2	Connector enhancer of kinase suppressor of ras 2 (Connector enhancer of KSR 2) (CNK homolog protein 2) (CNK2)	117,535		PTM: Phosphorylated on tyrosine
O95396	Adenylyltransferase and sulfurtransferase MOCS3 (Molybdenum cofactor synthesis protein 3) (Molybdopterin synthase sulfurylase) (MPT synthase sulfurylase) [Includes: Molybdopterin-synthase adenylyltransferase (EC 2.7.7.80) (Adenylyltransferase MOCS3) (Sulfur carrier protein MOCS2A adenylyltransferase); Molybdopterin-synthase sulfurtransferase (EC 2.8.1.11) (Sulfur carrier protein MOCS2A sulfurtransferase) (Sulfurtransferase MOCS3)]	49,669		
Q8IVN8	Somatomedin-B and thrombospondin type-1 domain-containing protein (RPE-spondin)	29,610	N-linked (GlcNAc...) asparagine	
P55055	Oxysterols receptor LXR-beta (Liver X receptor beta) (Nuclear receptor NER) (Nuclear receptor subfamily 1 group H member 2) (Ubiquitously-expressed nuclear receptor)	50,974		PTM: Sumoylated by SUMO2 at Lys-409 and Lys-447 during the hepatic acute phase response
Q7Z3I7	Zinc finger protein 572	61,238		
Q9Y580	RNA-binding protein 7 (RNA-binding motif protein 7)	30,504		PTM: Phosphorylated at Ser-136 by MAPK14/p38-alpha-activated MAPKAPK2/MK2
P48729	Casein kinase I isoform alpha (CKI-alpha) (EC 2.7.11.1) (CK1)	38,915		
P19532	Transcription factor E3 (Class E basic helix-loop-helix protein 33) (bHLHe33)	61,521		PTM: Phosphorylation by MTOR regulates its subcellular location and activity
Q8WXH4	Ankyrin repeat and SOCS box protein 11 (ASB-11)	35,367		
Q86U42	Polyadenylate-binding protein 2 (PABP-2) (Poly(A)-binding protein 2) (Nuclear poly(A)-binding protein 1) (Poly(A)-binding protein II) (PABII) (Polyadenylate-binding nuclear protein 1)	32,749		PTM: Arginine dimethylation is asymmetric and involves PRMT1 and PRMT3
Q96MT1	RING finger protein 145 (EC 2.3.2.27)	75,617		
P17643	5,6-dihydroxyindole-2-carboxylic acid oxidase (DHICA oxidase) (EC 1.14.18.-) (Catalase B) (Glycoprotein 75) (Melanoma antigen gp75) (Tyrosinase-related protein 1) (TRP) (TRP-1) (TRP1)	60,724	N-linked (GlcNAc...) asparagine	PTM: Glycosylated
Q9NW75	G patch domain-containing protein 2	58,944		
Q09472	Histone acetyltransferase p300 (p300 HAT) (EC 2.3.1.48) (E1A-associated protein p300) (Histone butyryltransferase p300) (EC 2.3.1.-) (Histone crotonyltransferase p300) (EC 2.3.1.-) (Protein 2-hydroxyisobutyryltransferase p300) (EC 2.3.1.-) (Protein lactyltransferas p300) (EC 2.3.1.-) (Protein propionyltransferase p300) (EC 2.3.1.-)	264,161		PTM: Acetylated on Lys at up to 17 positions by intermolecular autocatalysis
P58173	Olfactory receptor 2B6 (Hs6M1-32) (Olfactory receptor 2B1) (Olfactory receptor 2B5) (Olfactory receptor 5-40) (OR5-40) (Olfactory receptor 6-31) (OR6-31) (Olfactory receptor OR6-4)	35,414	N-linked (GlcNAc...) asparagine	
Q15776	Zinc finger protein with KRAB and SCAN domains 8 (LD5-1) (Zinc finger protein 192)	65,816		
Q9UHQ7	Transcription elongation factor A protein-like 9 (TCEA-like protein 9) (Transcription elongation factor S-II protein-like 9) (WW domain-binding protein 5) (WBP-5)	12,749		
P68402	Platelet-activating factor acetylhydrolase IB subunit alpha2 (EC 3.1.1.47) (PAF acetylhydrolase 30 kDa subunit) (PAF-AH 30 kDa subunit) (PAF-AH subunit beta) (PAFAH subunit beta)	25,569		
Q6PJF5	Inactive rhomboid protein 2 (iRhom2) (Rhomboid 5 homolog 2) (Rhomboid family member 2) (Rhomboid veinlet-like protein 5) (Rhomboid veinlet-like protein 6)	96,686		

Protein ID	Protein names	Mass	Glycosylation	Post-translational modification
Q8NB78	Lysine-specific histone demethylase 1B (EC 1.14.99.66) (Flavin-containing amine oxidase domain-containing protein 1) (Lysine-specific histone demethylase 2)	92,098		
Q2PZI1	Probable C-mannosyltransferase DPY19L1 (EC 2.4.1.-) (Dpy-19-like protein 1) (Protein dpy-19 homolog 1)	77,319		
A0A1B0G U71	Uncharacterized protein CFAP97D2 (CFAP97 domain-containing protein 2)	11,681		
P48745	CCN family member 3 (Cellular communication network factor 3) (Insulin-like growth factor-binding protein 9) (IBP-9) (IGF-binding protein 9) (IGFBP-9) (Nephro blastoma-overexpressed gene protein homolog) (Protein NOV homolog) (NovH)	39,162	N-linked (GlcNAc...) asparagine	PTM: May be palmitoylated on Cys-244, which is important for extracellular secretion
Q9NXW9	Alpha-ketoglutarate-dependent dioxygenase alkB homolog 4 (Alkylated DNA repair protein alkB homolog 4) (DNA N6-methyl adenine demethylase ALKBH4) (EC 1.14.11.51) (Lysine-specific demethylase ALKBH4) (EC 1.14.11.-)	33,838		
Q00G26	Perilipin-5 (Lipid storage droplet protein 5)	50,791		PTM: Phosphorylated by PKA. Phosphorylated on serine in skeletal muscle at rest or upon lipolytic stimulation.
P55895	V(D)J recombination-activating protein 2 (RAG-2)	59,241		
O95922	Probable tubulin polyglutamylase TTLL1 (EC 6.-.-.-) (Tubulin polyglutamylase complex subunit 3) (PGs3) (Tubulin--tyrosine ligase-like protein 1)	48,988		
A8MYP8	Outer dense fiber protein 3B (Outer dense fiber protein 3-like protein 3)	27,280		
Q9P2Y5	UV radiation resistance-associated gene protein (p63)	78,151		PTM: Phosphorylated at Ser-498 by MTOR under basal conditions
P28300	Protein-lysine 6-oxidase (EC 1.4.3.13) (Lysyl oxidase) [Cleaved into: Protein-lysine 6-oxidase, long form; Protein-lysine 6-oxidase, short form]	46,944	N-linked (GlcNAc...) asparagine	PTM: The lysine tyrosylquinone cross-link (LTQ) is generated by condensation of the epsilon-amino group of a lysine with a topaquinone produced by oxidation of tyrosine .
Q9BW92	Threonine--tRNA ligase, mitochondrial (EC 6.1.1.3) (Threonyl-tRNA synthetase) (ThrRS) (Threonyl-tRNA synthetase-like 1)	81,036		
Q86T96	E3 ubiquitin-protein ligase RNF180 (EC 2.3.2.27) (RING finger protein 180) (RING-type E3 ubiquitin transferase RNF180)	68,254		
P05000	Interferon omega-1 (Interferon alpha-II-1)	22,319	N-linked (GlcNAc...) asparagine	
P82663	28S ribosomal protein S25, mitochondrial (MRP-S25) (S25mt) (Mitochondrial small ribosomal subunit protein mS25)	20,116		
Q9UNQ2	Probable dimethyladenosine transferase (EC 2.1.1.183) (DIM1 dimethyladenosine transferase 1 homolog) (DIM1 dimethyladenosine transferase 1-like) (Probable 18S rRNA (adenine(1779)-N(6)/adenine(1780)-N(6))-dimethyltransferase) (Probable 18S rRNA dimethylase)	35,236		
Q12778	Forkhead box protein O1 (Forkhead box protein O1A) (Forkhead in rhabdomyosarcoma)	69,662		PTM: Phosphorylation by NLK promotes nuclear export and inhibits the transcriptional activity.
Q5T0L3	Spermatogenesis-associated protein 46	29,150		
Q9NYW1	Taste receptor type 2 member 9 (T2R9) (Taste receptor family B member 6) (TRB6)	35,611	N-linked (GlcNAc...) asparagine	
Q14D33	Receptor-transporting protein 5 (3CxxC-type zinc finger protein 5) (CXXC-type zinc finger protein 11)	60,488		

Protein ID	Protein names	Mass	Glycosylation	Post-translational modification
Q9BXI	TBC1 domain family member 10A (EBP50-PDX interactor of 64 kDa) (EPI64 protein) (Rab27A-GAP-alpha)	57,118		PTM: Exists in both phosphorylated and non-phosphorylated state.
A6NM28	Zinc finger protein 92 homolog (Zfp-92)	45,791		
P27797	Calreticulin (CRP55) (Calregulin) (Endoplasmic reticulum resident protein 60) (ERp60) (HACBP) (grp60)	48,142	N-linked (GlcNAc...) asparagine	
O95965	Integrin beta-like protein 1 (Osteoblast-specific cysteine-rich protein) (Ten integrin EGF-like repeat domain-containing protein)	53,921	N-linked (GlcNAc...) asparagine	
P49901	Sperm mitochondrial-associated cysteine-rich protein	12,767		
Q8TCD5	5'(3')-deoxyribonucleotidase, cytosolic type (EC 3.1.3.-) (Cytosolic 5',3'-pyrimidine nucleotidase) (Deoxy-5'-nucleotidase 1) (dNT-1)	23,383		
Q3MIR4	Cell cycle control protein 50B (P4-ATPase flippase complex beta subunit TMEM30B) (Transmembrane protein 30B)	38,941	N-linked (GlcNAc...) asparagine	
Q8TAG6	Vexin	22,584		
Q8NCW0	Kremen protein 2 (Dickkopf receptor 2) (Kringle domain-containing transmembrane protein 2) (Kringle-containing protein marking the eye and the nose)	48,849	N-linked (GlcNAc...) asparagine	
Q8N584	Tetratricopeptide repeat protein 39C (TPR repeat protein 39C)	65,870		
Q2HRB3	Capsid vertex component 2	61,424		
Q8WWX8	Sodium/myo-inositol cotransporter 2 (Na(+)/myo-inositol cotransporter 2) (Sodium-dependent glucose cotransporter) (Sodium/glucose cotransporter KST1) (Sodium/myo-inositol transporter 2) (SMIT2) (Solute carrier family 5 member 11)	74,036		
Q9NUQ3	Gamma-taxilin (Environmental lipopolysaccharide-responding gene protein) (Factor inhibiting ATF4-mediated transcription) (FIAT) (Lipopolysaccharide-specific response protein 5)	60,586		
Q8TEC5	E3 ubiquitin-protein ligase SH3RF2 (EC 2.3.2.27) (Heart protein phosphatase 1-binding protein) (HEPP1) (POSH-eliminating RING protein) (Protein phosphatase 1 regulatory subunit 39) (RING finger protein 158) (RING-type E3 ubiquitin transferase SH3RF2) (SH3 domain-containing RING finger protein 2)	79,320		PTM: Autoubiquitinated
Q9HAE3	EF-hand calcium-binding domain-containing protein 1	24,488		
Q15054	DNA polymerase delta subunit 3 (DNA polymerase delta subunit C) (DNA polymerase delta subunit p66) (DNA polymerase delta subunit p68)	51,400		PTM: Ubiquitinated
Q9NR12	PDZ and LIM domain protein 7 (LIM mineralization protein) (LMP) (Protein enigma)	49,845		
P0C7U0	Protein ELFN1 (Extracellular leucine-rich repeat and fibronectin type-III domain-containing protein 1) (Protein phosphatase 1 regulatory subunit 28)	90,477	N-linked (GlcNAc...) asparagine	
Q8N6M9	AN1-type zinc finger protein 2A	16,477		
Q9NVN3	Synembryn-B (Brain synembryn) (hSyn) (Protein Ric-8B)	58,825		
O75954	Tetraspanin-9 (Tspan-9) (Tetraspan NET-5)	26,779	N-linked (GlcNAc...) asparagine	PTM: Glycosylated
Q8IWA5	Choline transporter-like protein 2 (Solute carrier family 44 member 2)	80,124	N-linked (GlcNAc...) asparagine	
Q5PSV4	Breast cancer metastasis-suppressor 1-like protein (BRMS1-homolog protein p40) (BRMS1-like protein p40)	37,629		
Q96Q07	BTB/POZ domain-containing protein 9	69,188		

Protein ID	Protein names	Mass	Glycosylation	Post-translational modification
Q5VZR2	NUT family member 2G	79,011		
Q504Y0	Zinc transporter ZIP12 (LIV-1 subfamily of ZIP zinc transporter 8) (LZT-Hs8) (Solute carrier family 39 member 12) (Zrt- and Irt-like protein 12) (ZIP-12)	76,666		
P16455	Methylated-DNA--protein-cysteine methyltransferase (EC 2.1.1.63) (6-O-methylguanine-DNA methyltransferase) (MGMT) (O-6-methylguanine-DNA-alkyltransferase)	21,646		
Q4TWH8	Non-structural polyprotein 1AB [Cleaved into: VPg; Protein p19; Transmembrane protein 1A; Serine protease p27 (p27) (EC 3.4.21.-); Protein p20; RNA-directed RNA polymerase p57 (p57) (EC 2.7.7.48)]	161,728		
B1APH4	Putative zinc finger protein 487 (KRAB domain only protein 1)	51,624		
Q96CC6	Inactive rhomboid protein 1 (iRhom1) (Epidermal growth factor receptor-related protein) (Rhomboid 5 homolog 1) (Rhomboid family member 1) (p100hRho)	97,401	N-linked (GlcNAc...) asparagine	PTM: N-glycosylated.
Q9C0D3	Protein zyg-11 homolog B	83,921		
Q8TC27	Disintegrin and metalloproteinase domain-containing protein 32 (ADAM 32)	87,948	N-linked (GlcNAc...) asparagine	
P36873	Serine/threonine-protein phosphatase PP1-gamma catalytic subunit (PP-1G) (EC 3.1.3.16) (Protein phosphatase 1C catalytic subunit)	36,984		PTM: Phosphorylated by NEK2.
Q86SF2	N-acetylgalactosaminyltransferase 7 (EC 2.4.1.41) (Polypeptide GalNAc transferase 7) (GalNAc-T7) (pp-GaNTase 7) (Protein-UDP acetylgalactosaminyltransferase 7) (UDP-GalNAc:polypeptide N-acetylgalactosaminyltransferase 7)	75,389		
P59044	NACHT, LRR and PYD domains-containing protein 6 (Angiotensin II/vasopressin receptor) (PYRIN-containing APAF1-like protein 5)	98,768		
Q9NXS3	Kelch-like protein 28 (BTB/POZ domain-containing protein 5)	64,192		
Q8TD90	Melanoma-associated antigen E2 (Hepatocellular carcinoma-associated protein 3) (MAGE-E2 antigen)	60,378		
P23142	Fibulin-1 (FIBL-1)	77,214	N-linked (GlcNAc...) asparagine	
P40259	B-cell antigen receptor complex-associated protein beta chain (B-cell-specific glycoprotein B29) (Ig-beta) (Immunoglobulin-associated B29 protein) (CD antigen CD79b)	26,048	N-linked (GlcNAc...) asparagine	PTM: Phosphorylated on tyrosine upon B-cell activation.
Q53F39	Metallophosphoesterase 1 (EC 3.1.-.-) (Post-GPI attachment to proteins factor 5)	45,141		
O95858	Tetraspanin-15 (Tspan-15) (Tetraspan NET-7) (Transmembrane 4 superfamily member 15)	33,165	N-linked (GlcNAc...) asparagine	
P62995	Transformer-2 protein homolog beta (TRA-2 beta) (TRA2-beta) (hTRA2-beta) (Splicing factor, arginine/serine-rich 10) (Transformer-2 protein homolog B)	33,666		PTM: Phosphorylated in the RS domains.
P78317	E3 ubiquitin-protein ligase RNF4 (EC 2.3.2.27) (RING finger protein 4) (RING-type E3 ubiquitin transferase RNF4) (Small nuclear ring finger protein) (Protein SNURF)	21,319		PTM: Sumoylated; conjugated by one or two SUMO1 moieties.
Q14153	Protein FAM53B (Protein simplet)	45,768		
O94921	Cyclin-dependent kinase 14 (EC 2.7.11.22) (Cell division protein kinase 14) (Serine/threonine-protein kinase PFTAIRE-1) (hPFTAIRE1)	53,057		
Q00537	Cyclin-dependent kinase 17 (EC 2.7.11.22) (Cell division protein kinase 17) (PCTAIRE-motif protein kinase 2) (Serine/threonine-protein kinase PCTAIRE-2)	59,582		
Q8N398	von Willebrand factor A domain-containing protein 5B2	131,668		

Protein ID	Protein names	Mass	Glycosylation	Post-translational modification
Q16880	2-hydroxyacylsphingosine 1-beta-galactosyltransferase (EC 2.4.1.47) (Ceramide UDP-galactosyltransferase) (Cerebroside synthase) (UDP-galactose-ceramide galactosyltransferase)	61,438	N-linked (GlcNAc...) asparagine	
Q5TAP6	U3 small nucleolar RNA-associated protein 14 homolog C	87,188		
Q6XQN6	Nicotinate phosphoribosyltransferase (NAPRTase) (EC 6.3.4.21) (FHA-HIT-interacting protein) (Nicotinate phosphoribosyltransferase domain-containing protein 1)	57,578		PTM: Transiently phosphorylated on a His residue during the reaction cycle.
Q01130	Serine/arginine-rich splicing factor 2 (Protein PR264) (Splicing component, 35 kDa) (Splicing factor SC35) (SC-35) (Splicing factor, arginine/serine-rich 2)	25,476		PTM: Extensively phosphorylated on serine residues in the RS domain.
Q8TC56	Protein FAM71B	64,756		
Q9Y653	Adhesion G-protein coupled receptor G1 (G-protein coupled receptor 56) (Protein TM7XN1) [Cleaved into: ADGRG1 N-terminal fragment (ADGRG1 NT) (GPR56 N-terminal fragment) (GPR56 NT) (GPR56(N)) (GPR56 extracellular subunit) (GPR56 subunit alpha); ADGRG1 C-terminal fragment (ADGRG1 CT) (GPR56 C-terminal fragment) (GPR56 CT) (GPR56(C)) (GPR56 seven-transmembrane subunit) (GPR56 7TM) (GPR56 subunit beta)]	77,738	N-linked (GlcNAc...) asparagine	PTM: Autoproteolytically cleaved into 2 fragments; the large extracellular N-terminal fragment (ADGRG1 NT) and the membrane-bound C-terminal fragment (ADGRG1 CT) predominantly remain associated and non-covalently linked.
A6NGY5	Olfactory receptor 51F1	35,849		
Q6P047	Uncharacterized protein C8orf74	33,735		
O60902	Short stature homeobox protein 2 (Homeobox protein Og12X) (Paired-related homeobox protein SHOT)	34,953		
Q8NDV1	Alpha-N-acetylgalactosaminide alpha-2,6-sialyltransferase 3 (EC 2.4.99.7) (GalNAc alpha-2,6-sialyltransferase III) (ST6GalNAc III) (ST6GalNAcIII) (STY) (Sialyltransferase 7C) (SIAT7-C)	35,395	N-linked (GlcNAc...) asparagine	
Q6UXG8	Butyrophilin-like protein 9	59,716	N-linked (GlcNAc...) asparagine	
Q92772	Cyclin-dependent kinase-like 2 (EC 2.7.11.22) (Protein kinase p56 KKIAMRE) (Serine/threonine-protein kinase KKIAMRE)	56,019		
Q53EV4	Leucine-rich repeat-containing protein 23 (Leucine-rich protein B7)	39,761		
Q68CZ6	HAUS augmin-like complex subunit 3	69,650		
Q969W8	Zinc finger protein 566	49,219		
Q13480	GRB2-associated-binding protein 1 (GRB2-associated binder 1) (Growth factor receptor bound protein 2-associated protein 1)	76,616		PTM: Phosphorylated in response to FGFR1 activation. SH2 domains.
Q9UJT9	F-box/LRR-repeat protein 7 (F-box and leucine-rich repeat protein 7) (F-box protein FBL6/FBL7)	54,575		
Q8TD55	Pleckstrin homology domain-containing family O member 2 (PH domain-containing family O member 2) (Pleckstrin homology domain-containing family Q member 1) (PH domain-containing family Q member 1)	53,350		
Q92805	Golgin subfamily A member 1 (Golgin-97)	88,184		
O60268	Uncharacterized protein KIAA0513	46,639		
Q8N1Y9	Putative uncharacterized protein FLJ37218	24,915		
Q16581	C3a anaphylatoxin chemotactic receptor (C3AR) (C3a-R)	53,864	N-linked (GlcNAc...) asparagine	PTM: Among the sulfation sites Tyr-174 is essential for binding of C3a anaphylatoxin.
Q9NVP4	Double zinc ribbon and ankyrin repeat-containing protein 1	82,192		
P17029	Zinc finger protein with KRAB and SCAN domains 1 (Zinc finger protein 139) (Zinc finger protein 36) (Zinc finger protein KOX18)	63,630		

Protein ID	Protein names	Mass	Glycosylation	Post-translational modification
Q8IZ40	REST corepressor 2	58,012		
P62070	Ras-related protein R-Ras2 (EC 3.6.5.-) (Ras-like protein TC21) (Teratocarcinoma oncogene)	23,400		PTM: May be post-translationally modified by both palmitoylation and polyisoprenylation.
Q17RB0	Retrotransposon Gag-like protein 8B (Mammalian retrotransposon derived protein 8B)	13,246		
Q9Y5N6	Origin recognition complex subunit 6	28,107		
O43516	WAS/WASL-interacting protein family member 1 (Protein PRPL-2) (Wiskott-Aldrich syndrome protein-interacting protein) (WASP-interacting protein)	51,258		
Q9BY66	Lysine-specific demethylase 5D (EC 1.14.11.67) (Histocompatibility Y antigen) (H-Y) (Histone demethylase JARID1D) (Jumonji/ARID domain-containing protein 1D) (Protein SmcY) ([histone H3]-trimethyl-L-lysine(4) demethylase 5D)	174,073		
A1L190	Synaptonemal complex central element protein 3 (Testis highly expressed gene 2 protein) (THEG-2)	10,601		
P28324	ETS domain-containing protein Elk-4 (Serum response factor accessory protein 1) (SAP-1) (SRF accessory protein 1)	46,900		
A6NKC9	SH2 domain-containing protein 7	49,807		
Q9Y535	DNA-directed RNA polymerase III subunit RPC8 (RNA polymerase III subunit C8) (DNA-directed RNA polymerase III subunit H) (RNA polymerase III subunit 22.9 kDa subunit) (RPC22.9)	22,918		
Q969U6	F-box/WD repeat-containing protein 5 (F-box and WD-40 domain-containing protein 5)	63,922		PTM: Phosphorylated at Ser-151 by PLK4 during the G1/S transition, leading to inhibit its ability to ubiquitinate SASS6.
A0A0B4J1 X5	Immunoglobulin heavy variable 3-74	12,840		
P07196	Neurofilament light polypeptide (NF-L) (68 kDa neurofilament protein) (Neurofilament triplet L protein)	61,517	O-linked (GlcNAc) threonine	PTM: O-glycosylated.
P53367	Arfaptin-1 (ADP-ribosylation factor-interacting protein 1)	41,738		PTM: Phosphorylated by PRKD1
Q8IY34	Solute carrier family 15 member 3 (Osteoclast transporter) (Peptide transporter 3) (Peptide/histidine transporter 2)	63,560	N-linked (GlcNAc...) asparagine	
Q8IXL9	IQ domain-containing protein F2	19,627		
Q9BXW7	Haloacid dehalogenase-like hydrolase domain-containing 5 (Cat eye syndrome critical region protein 5)	46,321		
Q9BYV8	Centrosomal protein of 41 kDa (Cep41) (Testis-specific gene A14 protein)	41,368		
Q7KZI7	Serine/threonine-protein kinase MARK2 (EC 2.7.11.1) (EC 2.7.11.26) (ELKL motif kinase 1) (EMK-1) (MAP/microtubule affinity-regulating kinase 2) (PAR1 homolog) (PAR1 homolog b) (Par-1b) (Par1b)	87,911		PTM: Autophosphorylated
P05166	Propionyl-CoA carboxylase beta chain, mitochondrial (PCCase subunit beta) (EC 6.4.1.3) (Propanoyl-CoA:carbon dioxide ligase subunit beta)	58,216		
Q8NC60	Nitric oxide-associated protein 1	78,458		
Q8NGD4	Olfactory receptor 4K1 (Olfactory receptor OR14-19)	35,201	N-linked (GlcNAc...) asparagine	
Q9UKD2	mRNA turnover protein 4 homolog (Ribosome assembly factor MRTO4)	27,560		
Q9NV79	Protein-L-isoaspartate O-methyltransferase domain-containing protein 2	41,072		
Q92628	Uncharacterized protein KIAA0232	154,789		
P56705	Protein Wnt-4	39,052	N-linked (GlcNAc...) asparagine	PTM: Palmitoleoylation is required for efficient binding to frizzled receptors.

Protein ID	Protein names	Mass	Glycosylation	Post-translational modification
Q8IWB6	Inactive serine/threonine-protein kinase TEX14 (Protein kinase-like protein SgK307) (Sugen kinase 307) (Testis-expressed sequence 14) (Testis-expressed sequence 14 protein)	167,901		PTM: Phosphorylated on Thr residues by CDK1 during early phases of mitosis, promoting the interaction with PLK1 and recruitment to kinetochores.
Q9BYR2	Keratin-associated protein 4-5 (Keratin-associated protein 4.5) (Ultrahigh sulfur keratin-associated protein 4.5)	19,363		
Q6TDP4	Kelch-like protein 17 (Actinfilin)	69,874		
P18754	Regulator of chromosome condensation (Cell cycle regulatory protein) (Chromosome condensation protein 1)	44,969		PTM: N-terminal methylation by METTL11A/NTM1 is required for binding double-stranded DNA and stable chromatin association.
Q92796	Disks large homolog 3 (Neuroendocrine-DLG) (Synapse-associated protein 102) (SAP-102) (SAP102) (XLMR)	90,314		
Q9UNX9	ATP-sensitive inward rectifier potassium channel 14 (Inward rectifier K(+) channel Kir2.4) (IRK-4) (Potassium channel, inwardly rectifying subfamily J member 14)	47,846		
Q8N945	PRELI domain-containing protein 2	21,905		
Q8NGT0	Olfactory receptor 13C9 (Olfactory receptor OR9-13)	35,854	N-linked (GlcNAc...) asparagine	
P00749	Urokinase-type plasminogen activator (U-plasminogen activator) (uPA) (EC 3.4.21.73) [Cleaved into: Urokinase-type plasminogen activator long chain A; Urokinase-type plasminogen activator short chain A; Urokinase-type plasminogen activator chain B]	48,507	O-linked (Fuc) threonine	PTM: Phosphorylation of Ser-158 and Ser-323
Q8WZ19	BTB/POZ domain-containing adapter for CUL3-mediated RhoA degradation protein 1 (hBACURD1) (BTB/POZ domain-containing protein KCTD13) (Polymerase delta-interacting protein 1) (TNFAIP1-like protein)	36,357		
Q5TEU4	Arginine-hydroxylase NDUFAF5, mitochondrial (EC 1.-.-.-) (NADH dehydrogenase [ubiquinone] 1 alpha subcomplex assembly factor 5) (Putative methyltransferase NDUFAF5) (EC 2.1.1.-)	38,918		
P51687	Sulfite oxidase, mitochondrial (EC 1.8.3.1)	60,283		
Q8NEP9	Zinc finger protein 555	73,084		
P31749	RAC-alpha serine/threonine-protein kinase (EC 2.7.11.1) (Protein kinase B) (PKB) (Protein kinase B alpha) (PKB alpha) (Proto-oncogene c-Akt) (RAC-PK-alpha)	55,686	O-linked (GlcNAc) serine	PTM: O-GlcNAcylation at Thr-305 and Thr-312 inhibits activating phosphorylation at Thr-308 via disrupting the interaction between AKT1 and PDPK1.
P22090	40S ribosomal protein S4, Y isoform 1 (Small ribosomal subunit protein eS4)	29,456		
Q13410	Butyrophilin subfamily 1 member A1 (BT)	58,960	N-linked (GlcNAc...) asparagine	PTM: N-glycosylated
Q9UPY5	Cystine/glutamate transporter (Amino acid transport system xc-) (Calcium channel blocker resistance protein CCBR1) (Solute carrier family 7 member 11) (xCT)	55,423	N-linked (GlcNAc...) asparagine	
Q3SY46	Keratin-associated protein 13-3	19,236		
Q9BY84	Dual specificity protein phosphatase 16 (EC 3.1.3.16) (EC 3.1.3.48) (Mitogen-activated protein kinase phosphatase 7) (MAP kinase phosphatase 7) (MKP-7)	73,102		PTM: Phosphorylated at Ser-446 by MAPK1/ERK2, which prevents its degradation, and thereby stabilizes it and blocks JNK MAPK activity.
Q9H3N8	Histamine H4 receptor (H4R) (HH4R) (AXOR35) (G-protein coupled receptor 105) (GPRv53) (Pfi-013) (SP9144)	44,496	N-linked (GlcNAc...) asparagine	
Q9Y2L8	Zinc finger protein with KRAB and SCAN domains 5 (Zinc finger protein 95 homolog) (Zfp-95)	96,903		

Protein ID	Protein names	Mass	Glycosylation	Post-translational modification
Q9UKU7	Isobutyryl-CoA dehydrogenase, mitochondrial (IBDH) (EC 1.3.8.-) (Activator-recruited cofactor 42 kDa component) (ARC42) (Acyl-CoA dehydrogenase family member 8) (ACAD-8)	45,070		
Q96KP1	Exocyst complex component 2 (Exocyst complex component Sec5)	104,066		
Q7RTX7	Cation channel sperm-associated protein 4 (CatSper4)	54,092		
O00541	Pescadillo homolog	68,003		PTM: Sumoylated.
Q969M2	Gap junction alpha-10 protein (Connexin-62) (Cx62)	61,872		
Q8NFU5	Inositol polyphosphate multikinase (EC 2.7.1.140) (EC 2.7.1.151) (EC 2.7.1.153) (Inositol 1,3,4,6-tetrakisphosphate 5-kinase)	47,222		
Q9NQZ5	StAR-related lipid transfer protein 7, mitochondrial (Gestational trophoblastic tumor protein 1) (START domain-containing protein 7) (StARD7)	43,113		
Q96SZ6	Mitochondrial tRNA methylthiotransferase CDK5RAP1 (EC 2.8.4.3) (CDK5 activator-binding protein C42) (CDK5 regulatory subunit-associated protein 1) (mt-tRNA-2-methylthio-N6-dimethylallyladenosine synthase) (mt-tRNA-N6-(dimethylallyl)adenosine(37) methylthiotransferase)	67,689		
O75570	Peptide chain release factor 1, mitochondrial (MRF-1) (MtRF-1)	52,306		
Q15428	Splicing factor 3A subunit 2 (SF3a66) (Spliceosome-associated protein 62) (SAP 62)	49,256		
O15151	Protein Mdm4 (Double minute 4 protein) (Mdm2-like p53-binding protein) (Protein Mdmx) (p53-binding protein Mdm4)	54,864		PTM: Phosphorylated.
P09683	Secretin	13,016		
P19237	Troponin I, slow skeletal muscle (Troponin I, slow-twitch isoform)	21,692		
Q96RT6	cTAGE family member 2 (Protein cTAGE-2) (Cancer/testis antigen 21.2) (CT21.2)	85,282		
Q9H4A6	Golgi phosphoprotein 3 (Coat protein GPP34) (Mitochondrial DNA absence factor) (MIDAS)	33,811		PTM: Phosphorylated
A5D8V6	Vacuolar protein sorting-associated protein 37C (hVps37C) (ESCRT-I complex subunit VPS37C)	38,659		PTM: Phosphorylated by TBK1.
Q7Z4P5	Growth/differentiation factor 7 (GDF-7)	46,950	N-linked (GlcNAc...) asparagine	
Q6FHJ7	Secreted frizzled-related protein 4 (sFRP-4) (Frizzled protein, human endometrium) (FrpHE)	39,827	N-linked (GlcNAc...) asparagine	
Q8N782	Zinc finger protein 525	23,070		
Q6IE37	Ovostatin homolog 1	134,499	N-linked (GlcNAc...) asparagine	
Q99895	Chymotrypsin-C (EC 3.4.21.2) (Caldecrin)	29,484	N-linked (GlcNAc...) asparagine	
Q9H4Q3	PR domain zinc finger protein 13 (EC 2.1.1.-) (PR domain-containing protein 13)	73,981		
Q9NQX3	Gephyrin [Includes: Molybdopterin adenylyltransferase (MPT adenylyltransferase) (EC 2.7.7.75) (Domain G); Molybdopterin molybdenumtransferase (MPT Mo-transferase) (EC 2.10.1.1) (Domain E)]	79,748		PTM: Palmitoylated ECO:0000269|PubMed:25025157}.
O43734	E3 ubiquitin ligase TRAF3IP2 (EC 2.3.2.27) (Adapter protein CIKS) (Connection to IKK and SAPK/JNK) (E3 ubiquitin-protein ligase CIKS) (Nuclear factor NF-kappa-B activator 1) (ACT1) (TRAF3-interacting protein 2)	64,666		
Q9H816	5' exonuclease Apollo (EC 3.1.-.-) (DNA cross-link repair 1B protein) (SNM1 homolog B) (SNMIB) (hSNM1B)	60,002		PTM: Ubiquitinated, leading to its degradation. Interaction with TERF2 protects it from ubiquitination.

Protein ID	Protein names	Mass	Glycosylation	Post-translational modification
Q96C01	Protein FAM136A	15,641		
C9JC47	Putative protein FAM157A	42,896		
Q7Z589	BRCA2-interacting transcriptional repressor EMSY	141,468	O-linked (GlcNAc) serine	PTM: O-glycosylated during cytokinesis at sites identical or close to phosphorylation sites, this interferes with the phosphorylation status.
Q9HBD1	Roquin-2 (EC 2.3.2.27) (Membrane-associated nucleic acid-binding protein) (RING finger and CCCH-type zinc finger domain-containing protein 2) (RING finger protein 164) (RING-type E3 ubiquitin transferase Roquin-2)	131,669		PTM: Proteolytically cleaved after Arg-509 and Arg-585 by MALT1 in activated CD4(+) T cells
O15116	U6 snRNA-associated Sm-like protein LSm1 (Cancer-associated Sm-like) (Small nuclear ribonuclear CaSm)	15,179		
P23193	Transcription elongation factor A protein 1 (Transcription elongation factor S-II protein 1) (Transcription elongation factor TFIIS.o)	33,970		
Q5SZD4	Glycine N-acyltransferase-like protein 3 (EC 2.3.1.13) (Acyl-CoA:glycine-N-acyltransferase-like protein 3)	32,704		
Q9UBM7	7-dehydrocholesterol reductase (7-DHC reductase) (EC 1.3.1.21) (Delta7-sterol reductase) (Sterol Delta(7)-reductase) (Sterol reductase SR-2)	54,489		
Q9UMR7	C-type lectin domain family 4 member A (C-type lectin DDB27) (C-type lectin superfamily member 6) (Dendritic cell immunoreceptor) (Lectin-like immunoreceptor) (CD antigen CD367)	27,512	N-linked (GlcNAc...) asparagine	
Q92859	Neogenin (Immunoglobulin superfamily DCC subclass member 2)	160,017	N-linked (GlcNAc...) asparagine	
Q96H12	Myb/SANT-like DNA-binding domain-containing protein 3	32,363		
Q9BV35	Calcium-binding mitochondrial carrier protein SCaMC-3 (Mitochondrial ATP-Mg/Pi carrier protein 2) (Mitochondrial Ca(2+)-dependent solute carrier protein 2) (Small calcium-binding mitochondrial carrier protein 3) (Solute carrier family 25 member 23)	52,378		
Q13610	Periodic tryptophan protein 1 homolog (Keratinocyte protein IEF SSP 9502)	55,828		
Q5T1N1	Protein AKNAD1	92,864		
Q4VC05	B-cell CLL/lymphoma 7 protein family member A	22,810		
Q9BV44	THUMP domain-containing protein 3	57,003		
O00622	CCN family member 1 (Cellular communication network factor 1) (Cysteine-rich angiogenic inducer 61) (Insulin-like growth factor-binding protein 10) (IBP-10) (IGF-binding protein 10) (IGFBP-10) (Protein CYR61) (Protein GIG1)	42,027		
Q9NSA0	Solute carrier family 22 member 11 (Organic anion transporter 4)	59,972	N-linked (GlcNAc...) asparagine	PTM: N-glycosylated. Contains several complex-type N-glycans
O75063	Glycosaminoglycan xylosylkinase (EC 2.7.1.-) (Xylose kinase)	46,432	N-linked (GlcNAc...) asparagine	
Q8N635	Meiosis-specific with OB domain-containing protein (EC 3.1.-.-)	49,313		
Q8WV44	E3 ubiquitin-protein ligase TRIM41 (EC 2.3.2.27) (RING finger-interacting protein with C kinase) (RINCK) (RING-type E3 ubiquitin transferase TRIM41) (Tripartite motif-containing protein 41)	71,670		PTM: Auto-ubiquitinated.
Q15435	Protein phosphatase 1 regulatory subunit 7 (Protein phosphatase 1 regulatory subunit 22)	41,564		
Q8N7R1	POM121-like protein 12	31,848		

Protein ID	Protein names	Mass	Glycosylation	Post-translational modification
Q9UH90	F-box only protein 40 (Muscle disease-related protein)	79,782		
Q6ZSS3	Zinc finger protein 621	49,205		
Q5R3I4	Tetratricopeptide repeat protein 38 (TPR repeat protein 38)	52,787		
P30837	Aldehyde dehydrogenase X, mitochondrial (EC 1.2.1.3) (Aldehyde dehydrogenase 5) (Aldehyde dehydrogenase family 1 member B1)	57,206		
Q68DY1	Zinc finger protein 626	60,893		
A6NES4	Maestro heat-like repeat-containing protein family member 2A (HEAT repeat-containing protein 7B1)	189,561		
Q96PP8	Guanylate-binding protein 5 (EC 3.6.5.-) (GBP-TA antigen) (GTP-binding protein 5) (GBP-5) (Guanine nucleotide-binding protein 5)	66,617		PTM: Isoprenylation is required for proper subcellular location.
Q8TC71	Mitochondria-eating protein (Spermatogenesis-associated protein 18)	61,109		
P19883	Follistatin (FS) (Activin-binding protein)	38,007	N-linked (GlcNAc...) asparagine	
Q9UPU9	Protein Smaug homolog 1 (Smaug 1) (hSmaug1) (Sterile alpha motif domain-containing protein 4A) (SAM domain-containing protein 4A)	79,415		
Q6IS24	Polypeptide N-acetylgalactosaminyltransferase 17 (EC 2.4.1.41) (Polypeptide GalNAc transferase-like protein 3) (GalNAc-T-like protein 3) (pp-GaNTase-like protein 3) (Protein-UDP acetylgalactosaminyltransferase-like protein 3) (UDP-GalNAc:polypeptide N-acetylgalactosaminyltransferase-like protein 3) (Williams-Beuren syndrome chromosomal region 17 protein)	67,751	N-linked (GlcNAc...) asparagine	
Q96SL8	Flt3-interacting zinc finger protein 1 (Zinc finger protein 798)	51,996		
Q9Y283	Inversin (Inversion of embryo turning homolog) (Nephrocystin-2)	117,826		PTM: May be ubiquitinated via its interaction with APC2.
Q03111	Protein ENL (YEATS domain-containing protein 1)	62,056		
Q9H5Y7	SLIT and NTRK-like protein 6	95,110		
Q9BRP7	Ferredoxin-fold anticodon-binding domain-containing protein 1 (FDX-ACDB domain-containing protein 1)	70,416		
Q63HQ2	Pikachurin (Agrin-like protein) (EGF-like, fibronectin type-III and laminin G-like domain-containing protein)	111,271	N-linked (GlcNAc...) asparagine	PTM: O-glycosylated; contains chondroitin sulfate and heparan sulfate.
Q8WTV1	THAP domain-containing protein 3	27,059		
O94907	Dickkopf-related protein 1 (Dickkopf-1) (Dkk-1) (hDkk-1) (SK)	28,672	O-linked (GlcNAc) serine	
Q13884	Beta-1-syntrophin (59 kDa dystrophin-associated protein A1 basic component 1) (DAPA1B) (BSYN2) (Syntrophin-2) (Tax interaction protein 43) (TIP-43)	58,061		PTM: Phosphorylated by CaM-kinase II
P34059	N-acetylgalactosamine-6-sulfatase (EC 3.1.6.4) (Chondroitinsulfatase) (Chondroitinase) (Galactose-6-sulfate sulfatase) (GalN6S) (N-acetylgalactosamine-6-sulfate sulfatase) (GalNAc6S sulfatase)	58,026	N-linked (GlcNAc...) asparagine	PTM: The conversion to 3-oxoalanine (also known as C-formylglycine, FGly), of a serine or cysteine residue in prokaryotes and of a cysteine residue in eukaryotes, is critical for catalytic activity.
Q6GMR7	Fatty-acid amide hydrolase 2 (EC 3.5.1.99) (Amidase domain-containing protein) (Anandamide amidohydrolase 2) (Oleamide hydrolase 2)	58,304		
Q9ULB5	Cadherin-7	87,086	N-linked (GlcNAc...) asparagine	
Q96L33	Rho-related GTP-binding protein RhoV (CDC42-like GTPase 2) (GTP-binding protein-like 2) (Rho GTPase-like protein ARHV) (Wnt-1 responsive Cdc42 homolog 2) (WRCH-2)	26,217		

Protein ID	Protein names	Mass	Glycosylation	Post-translational modification
Q8NGI2	Olfactory receptor 52N4 (Olfactory receptor OR11-64)	36,080	N-linked (GlcNAc...) asparagine	
P19022	Cadherin-2 (CDw325) (Neural cadherin) (N-cadherin) (CD antigen CD325)	99,809	N-linked (GlcNAc...) asparagine	PTM: Cleaved by MMP24
Q9NQ11	Polyamine-transporting ATPase 13A2 (EC 7.6.2.-)	128,794	N-linked (GlcNAc...) asparagine	PTM: Autophosphorylated
P60852	Zona pellucida sperm-binding protein 1 (Zona pellucida glycoprotein 1) (Zp-1) [Cleaved into: Processed zona pellucida sperm-binding protein 1]	70,049	N-linked (GlcNAc...) asparagine	
O14863	Zinc transporter 4 (ZnT-4) (Solute carrier family 30 member 4)	47,483		
Q6P0Q8	Microtubule-associated serine/threonine-protein kinase 2 (EC 2.7.11.1)	196,436		PTM: Phosphorylated and ubiquitinated
Q53EU6	Glycerol-3-phosphate acyltransferase 3 (GPAT-3) (EC 2.3.1.15) (1-acyl-sn-glycerol-3-phosphate O-acyltransferase 10) (AGPAT 10) (1-acyl-sn-glycerol-3-phosphate O-acyltransferase 9) (1-AGP acyltransferase 9) (1-AGPAT 9) (EC 2.3.1.51) (Acyl-CoA:glycerol-3-phosphate acyltransferase 3) (hGPAT3) (Lung cancer metastasis-associated protein 1) (Lysophosphatidic acid acyltransferase theta) (LPAAT-theta) (MAG-1)	48,705		
Q9H4A4	Aminopeptidase B (AP-B) (EC 3.4.11.6) (Arginine aminopeptidase) (Arginyl aminopeptidase)	72,596		
P12821	Angiotensin-converting enzyme (ACE) (EC 3.2.1.-) (EC 3.4.15.1) (Dipeptidyl carboxypeptidase I) (Kininase II) (CD antigen CD143) [Cleaved into: Angiotensin-converting enzyme, soluble form]	149,715	N-linked (GlcNAc...) asparagine	PTM: Phosphorylated by CK2 on Ser-1299; which allows membrane retention.
Q9H6U8	Alpha-1,2-mannosyltransferase ALG9 (EC 2.4.1.259) (EC 2.4.1.261) (Asparagine-linked glycosylation protein 9 homolog) (Disrupted in bipolar disorder protein 1) (Dol-P-Man:Man(6)GlcNAc(2)-PP-Dol alpha-1,2-mannosyltransferase) (Dol-P-Man:Man(8)GlcNAc(2)-PP-Dol alpha-1,2-mannosyltransferase)	69,863	N-linked (GlcNAc...) asparagine	
Q7Z6G8	Ankyrin repeat and sterile alpha motif domain-containing protein 1B (Amyloid-beta protein intracellular domain-associated protein 1) (AIDA-1) (E2A-PBX1-associated protein) (EB-1)	138,066		PTM: Isoform 3 nuclear translocation requires an NMDAR-dependent proteolytic cleavage.
Q9Y2Z9	Ubiquinone biosynthesis monooxygenase COQ6, mitochondrial (EC 1.14.13.-) (Coenzyme Q10 monooxygenase 6)	50,870		
Q12906	Interleukin enhancer-binding factor 3 (Double-stranded RNA-binding protein 76) (DRBP76) (M-phase phosphoprotein 4) (MPP4) (Nuclear factor associated with dsRNA) (NFAR) (Nuclear factor of activated T-cells 90 kDa) (NF-AT-90) (Translational control protein 80) (TCP80)	95,338		
Q9GZN2	Homeobox protein TGIF2 (5'-TG-3'-interacting factor 2) (TGF-beta-induced transcription factor 2) (TGFB-induced factor 2)	25,878		PTM: The C-terminal part is phosphorylated in response to EGF signaling by the Ras/MAPK pathway.
Q8N0Z6	Tetratricopeptide repeat protein 5 (TPR repeat protein 5) (Stress-responsive activator of p300) (Strap)	48,928		PTM: Phosphorylation at Ser-203 enhances protein stability, regulates nuclear accumulation and association with p300/EP300.
